# Unusually High Incidences of *Staphylococcus aureus* Infection within Studies of Ventilator Associated Pneumonia Prevention Using Topical Antibiotics: Benchmarking the Evidence Base

**DOI:** 10.3390/microorganisms6010002

**Published:** 2018-01-04

**Authors:** James C. Hurley

**Affiliations:** 1 Rural Health Academic Center, Melbourne Medical School, University of Melbourne, 3350 Ballarat, Australia; jamesh@bhs.org.au; Tel.: +61-3-5320-4322; 2 Division of Internal Medicine, Ballarat Health Services, 3350 Ballarat, VIC, Australia

**Keywords:** ventilator associated pneumonia, *Staphylococcus aureus*, antibiotic prophylaxis, study design, intensive care, mechanical ventilation, cross infection, nosocomial infection, selective digestive decontamination, antibiotics

## Abstract

Selective digestive decontamination (SDD, topical antibiotic regimens applied to the respiratory tract) appears effective for preventing ventilator associated pneumonia (VAP) in intensive care unit (ICU) patients. However, potential contextual effects of SDD on *Staphylococcus aureus* infections in the ICU remain unclear. The *S. aureus* ventilator associated pneumonia (*S. aureus* VAP), VAP overall and *S. aureus* bacteremia incidences within component (control and intervention) groups within 27 SDD studies were benchmarked against 115 observational groups. Component groups from 66 studies of various interventions other than SDD provided additional points of reference. In 27 SDD study control groups, the mean *S. aureus* VAP incidence is 9.6% (95% CI; 6.9–13.2) versus a benchmark derived from 115 observational groups being 4.8% (95% CI; 4.2–5.6). In nine SDD study control groups the mean *S. aureus* bacteremia incidence is 3.8% (95% CI; 2.1–5.7) versus a benchmark derived from 10 observational groups being 2.1% (95% CI; 1.1–4.1). The incidences of *S. aureus* VAP and *S. aureus* bacteremia within the control groups of SDD studies are each higher than literature derived benchmarks. Paradoxically, within the SDD intervention groups, the incidences of both *S. aureus* VAP and VAP overall are more similar to the benchmarks.

## 1. Introduction

Ventilator associated pneumonia (VAP) in association with *Staphylococcus aureus* has been reported in numerous studies from intensive care units (ICU) worldwide [1,2,3,4,5,6,7,8,9,10,11,12,13,14,15,16,17,18,19,20,21,22,23,24,25,26,27,28,29,30,31,32,33,34,35,36,37,38,39,40,41,42,43,44,45,46,47,48,49,50,51,52,53,54,55,56,57,58,59,60,61,62,63,64,65,66,67,68,69,70,71,72,73,74,75,76,77,78,79,80,81,82,83,84,85,86,87,88,89,90,91,92,93,94,95,96,97,98,99,100,101,102,103,104,105,106,107,108,109,110,111,112,113,114,115,116,117,118,119,120,121,122,123,124,125,126,127,128,129,130,131,132,133,134,135,136,137,138,139,140,141,142,143,144,145,146,147,148,149,150,151,152,153,154,155,156,157,158,159,160,161,162,163,164,165,166,167,168,169,170,171,172,173,174,175,176,177,178,179,180,181,182,183,184,185,186,187,188,189,190,191,192,193,194,195,196]. Approximately 20% of ICU patients receiving prolonged mechanical ventilation develop ventilator-associated pneumonia (VAP) and *S. aureus* accounts for approximately 20% of VAP isolates [197]. 

Selective digestive decontamination (SDD) is a novel intervention using various regimens of topical antibiotics that are applied to the upper airway of patients receiving mechanical ventilation to reduce microbial colonization with the objective of preventing not only pneumonia but also bacteremic infections [198,199,200,201,202]. In contrast to other infection prevention strategies such as topical antiseptics such as chlorhexidine or non-antimicrobial methods, SDD selectively decreases colonization with aerobic gram negative bacilli at the oro-pharynx. However, SDD may increase colonization with gram positive bacteria including staphylococci [203,204]. 

In numerous studies and systematic reviews, the evidence in support of SDD in preventing ICU acquired infections such as VAP and bacteremia, appears compelling. These studies and systematic reviews report apparent reductions in the overall incidence of VAP of >50% [198,199,200,201,202] versus other methods of VAP prevention for which the reduction is generally <50% [205,206,207,208,209,210,211,212,213,214,215,216,217,218]. An apparent reduction in the overall incidence of bacteremia of >25% with SDD is also reported [199,200].

The effects of topical antibiotics such as SDD on the incidence of acquired infection with *S. aureus* in the ICU is of interest for two reasons.

Firstly, there is a concern that decontamination regimens might alter the ICU microbiome and the infection risk for those patients concurrent in the ICU not receiving the intervention [219,220,221,222]. Where any contextual risk leads to decreases or increases in infections rates it is termed “herd protection” and “herd peril”, respectively [223]. Typically, contextual effects are not apparent within individual studies examined in isolation but become apparent only by comparing the infection incidences observed within the individual studies to incidence benchmarks derived from other studies in the literature. 

Second, it is possible that the effect of topical placebo in this context is not neutral in that the proportion of *Staphylococcus aureus* among VAP isolates is higher among studies of SDD that used topical placebo to achieve observer blinding than among studies without any intervention [224]. Clarifying whether these contextual or placebo effects might be present within studies of VAP prevention methods is crucial towards evaluating any apparent effect sizes of SDD [225].

The overall study objectives here are

- to survey and visually compare the incidence of *S. aureus* VAP (and VAP overall) within component (control and intervention) groups decanted from these studies versus an external benchmark.
- to model the effects of various group level factors within these studies on *S. aureus* VAP (and VAP overall) incidence. A key factor is membership of a component group of a study which either did or did not use topical placebo to achieve observer blinding.
- To collate data on the incidence of *S. aureus* bacteremia and methicillin resistant *S. aureus* (MRSA) VAP among those studies for which this data is available.

In all three objectives, a category of observational studies serves to derive external benchmarks for the incidences of *S. aureus* VAP, MRSA-VAP, VAP overall and *S. aureus* bacteremia. Also, studies of interventions other than topical antibiotics, being studies of non-antibiotic methods and studies of methods using topical antiseptics, provide additional points of reference. 

## 2. Materials and Methods

Being an analysis of published work, ethics committee review of this study was not required.

### 2.1. Study Selection and Decant of Groups

**Figure 1:**
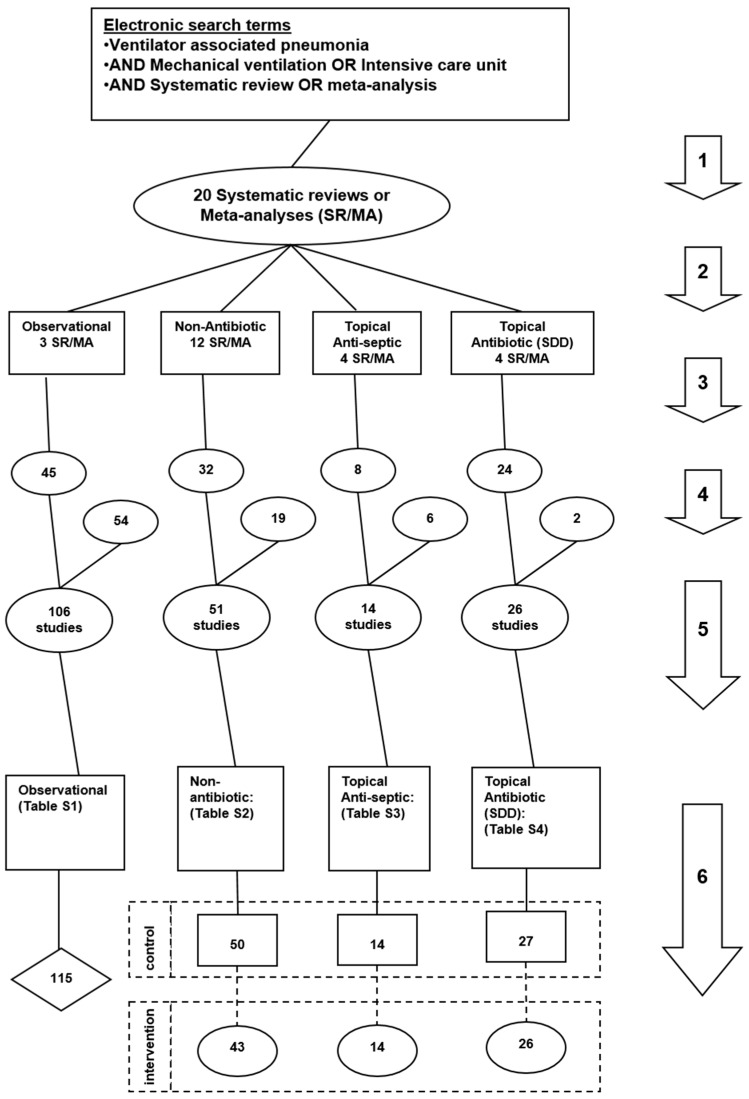
Flow chart of literature search and study and group decant. Search method, screening criteria and resulting classification of eligible studies and subsequent decant of component groups. The six numbered arrows are as follows; (**1**) An electronic search for systematic reviews containing potentially eligible studies using search terms; “ventilator associated pneumonia”, “mechanical ventilation”, “intensive care unit”, each combined with either “meta-analysis” or “systematic review” up to December 2015; (**2**) Studies were streamed into one of four categories; studies in which there was no intervention (observational studies), studies of non-antibiotic methods, topical antiseptics or topical antibiotics; (**3**) The studies were screened against inclusion and exclusion criteria; (**4**) A hand search was undertaken for additional studies; (**5**) eligible studies were then collated and any duplicate studies were removed; (**6**) The component groups were decanted from each study being control (rectangles), intervention (ovals) and observation (diamond) groups. Note; the total numbers do not tally as some systematic reviews provided studies in more than one category and some studies provided groups in more than one category.

The literature search and analytic approach used here (Figure 1) is as described previously [223]. These six steps (Figure 1; numbered arrows) are as follows:

1. An electronic search of PubMed, The Cochrane database and Google Scholar for systematic reviews containing potentially eligible studies was undertaken using the following search terms; “ventilator associated pneumonia”, “mechanical ventilation”, “intensive care unit”, each combined with either “meta-analysis” or “systematic review” up to December 2015. The use of systematic reviews as the starting point for benchmarking *S. aureus* VAP incidence serves two purposes; they provide estimates of the apparent effect size of the interventions of interest and, they provide objective and transparent sources of VAP incidence data.
2. Systematic reviews of studies of patient populations requiring prolonged (>24 h) mechanical ventilation were then streamed into one of three categories; systematic reviews of studies in which there was no intervention (observational studies), systematic reviews of infection prevention studies using topical antibiotics in any formulation [198,199,200,201,202], systematic reviews of studies of non-antibiotic interventions (non-antibiotic studies) and systematic reviews of studies of topical antiseptics. The studies of non-antibiotic methods of VAP prevention encompass a broad range of methods delivered either via the gastric route [205,206,207,208], the airway route [209,210,211,212,213,214,215,216] or via the oral care route [201,202,217,218].
3. The studies within these systematic reviews were screened against the following eligibility criteria. Inclusion criteria; infection prevention studies using concurrent controls and also observational studies for which incidence data for *S. aureus* VAP was extractable as an incidence proportion. The denominator for this incidence proportion is the numbers of patients receiving mechanical ventilation with an ICU stay of at least 24 h. Exclusion criteria; studies limited to patients with the acute respiratory distress syndrome. Studies in a language other than English were included where these had been abstracted in an English language systematic review.
4. A hand search was undertaken for additional studies not identified within systematic reviews including studies published since 2015.
5. All eligible studies were then collated and any duplicate studies were removed and streamed into groups of patients from studies without a VAP prevention method (observational groups) or component groups of the studies of antibiotics, studies of anti-septics and studies of non-antibiotic interventions.
6. The component groups were decanted from each study as observational, control or intervention groups.

### 2.2. Outcomes of Interest

The *S. aureus* VAP proportion is derived as follows; the numerator is the number of patients with VAP found to have an *S. aureus* isolate and the denominator is the number of patients receiving prolonged mechanical ventilation. In addition, the following were also extracted where available; the proportion of admissions for trauma, the incidence proportion of VAP overall, the incidence proportion of MRSA-VAP’s, and the incidence proportion of *S. aureus* bacteremia. Those groups for which the proportion of admissions for trauma was >50% were arbitrarily designated as originating from trauma ICU’s. The bacteremia incidences were expressed as a proportion using the number of patients with prolonged (>24 h) mechanical ventilation in the ICU as the denominator. Other parameters extracted were whether the mode of VAP diagnosis required bronchoscopic sampling, and whether the study originated from either the United States of America or Canada (North America).

### 2.3. Benchmarking: Visual

Scatter plots were generated to facilitate a visual benchmark of the VAP, *S. aureus* VAP and bacteremia incidence rates and these were generated as follows. The data for VAP, *S. aureus* VAP, overall bacteremia and *S. aureus* bacteremia, were logit transformed as previously [27]. For *S. aureus* VAP this transformation proceeds as follows; with the number of mechanically ventilated patients as the denominator (D), the number of patients with *S. aureus* VAP as the numerator (N), and R being the *S. aureus* VAP proportion (N/D), the logit (*S. aureus* VAP) is log(N/(D − N)) and its variance is 1/(D × R × (1 − R)). The *S. aureus* VAP benchmark is derived using the observational studies and is the mean of the logit (*S. aureus* VAP) weighted by the inverse variance. The derived logits were back transformed onto the percentage scale. The overall VAP and *S. aureus* bacteremia benchmarks were likewise derived. For each end point, the benchmark is the mean incidence derived from the observational studies. These respective benchmarks were then used as reference lines in the scatterplots of *S. aureus* VAP, VAP overall, MRSA-VAP, and bacteremia. 

### 2.4. Benchmarking: Meta-Regression

Group level regression models of VAP and *S. aureus* VAP proportions were developed using generalized estimating equation methods (‘xtgee’ command in STATA; release 12.0, STATA Corp., College Station, TX, USA). Generalized estimating equation regression models accommodate any intra-cluster correlation. In these regression models, the predictor variables were the component group membership as follows; type of component group being either a control or intervention and type of intervention under study being a topical antibiotic, a topical antiseptic or a non-antibiotic intervention. Additional predictor variables were; origin from a trauma ICU, origin from a North American ICU, whether the mode of diagnosis of VAP required bronchoscopic sampling and year of study publication. All factors in both models were entered as discrete variables with the category of observational groups acting as the reference (benchmark) category in each model. All factors were entered into the regression models without any pre-selection step.

## 3. Results

### 3.1. Characteristics of the Studies

Of the 198 studies identified by the search (Figure 1), 109 were sourced from 19 systematic reviews (Table 1) [198,199,200,201,202,205,206,207,208,209,210,211,212,213,214,215,216,217,218]. The majority of studies were published between 1990 and 2010 and a minority originated from trauma ICU’s. Bronchoscopic methods for VAP sampling and diagnosis were more commonly used among observational studies than other studies. The studies of non-antibiotic based methods were drawn from studies of gastric acid based and airway based interventions for the prevention of ICU acquired infections among patients receiving prolonged mechanical ventilation. The studies of topical antiseptic based methods include various interventions such as chlorhexidine and povidone-iodine as topical antiseptic agents [157,158,159,160,161,162,163,164,165,166,167,168,169,170]. There were 27 studies of topical antibiotics which studied various types of SDD and similar topical antibiotic interventions. 

A total of 289 component groups were decanted from these 198 studies. There were 115 groups from observational studies Table S1, see Additional file 1), 93 groups from studies of various non-antibiotic based methods of VAP prevention (Table S2, see Additional file 1), 28 groups from studies of topical anti-septics and 53 groups from studies of topical antibiotics (Table S3, see Additional file 1). Twelve studies had more than one observational, control or intervention group. The majority of groups from studies of topical antibiotics methods had less than 60 patients per group versus more than 70 patients in the majority of all remaining groups.

### 3.2. VAP Benchmarking: Visual

**Figure 2:**
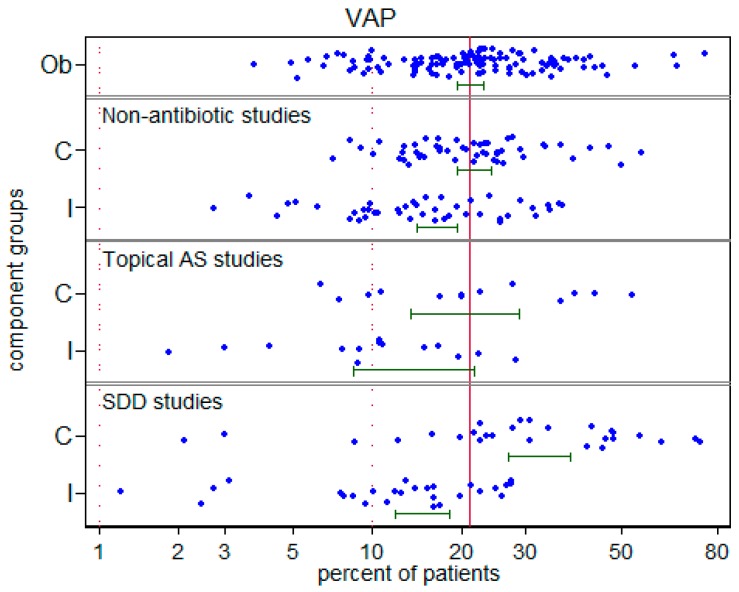
Incidence of ventilator associated pneumonia (VAP) versus benchmark. The component (C = control; I = intervention) groups of studies in which there was no intervention (Ob = observational), studies of non-antibiotic methods, topical antiseptics (AS) or topical antibiotics (SDD). The VAP benchmark is the summary mean (central vertical line) derived from the observation studies. These data are listed in Table S1–S4 (see Additional file 1). Note that the horizontal axis is a logit scale.

**Figure 3:**
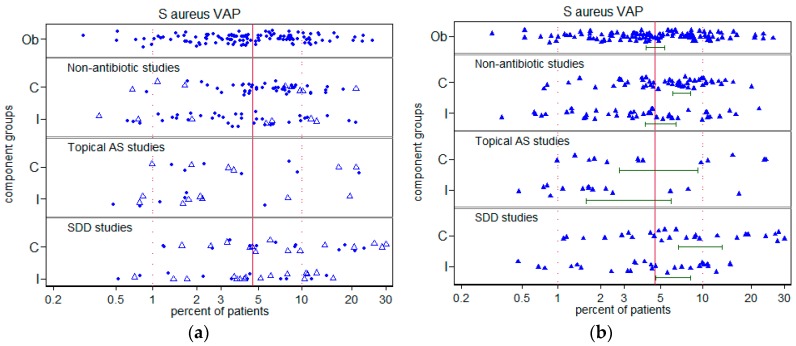
Incidence of *S. aureus* VAP versus benchmark. The component (C = control; I = intervention) groups of studies in which there was no intervention (Ob = observational), studies of non-antibiotic methods, topical antiseptics (AS) or topical antibiotics (SDD). The *S. aureus* VAP benchmark is the summary mean (central vertical line) derived from the observation studies (These data are listed in Table S1–S4 (see Additional file 1). Note that the horizontal axis is a logit scale. Figure 3**a** (top) indicates component groups that came studies that were (open triangles) or were not (solid triangles) from studies in which topical placebo was used to achieve observer blinding and Figure 3**b** (bottom) indicates the mean incidence (and 95% confidence interval) for each category.

**Figure 4:**
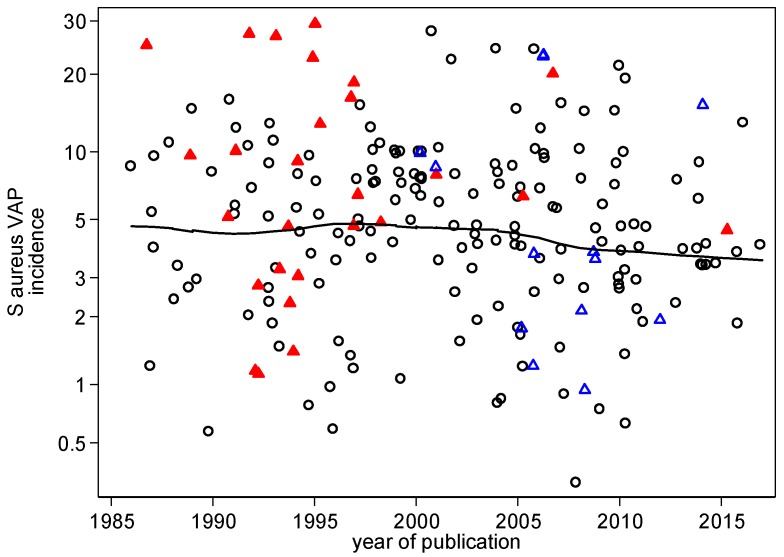
*S. aureus* VAP incidence versus year of publication. Dot plot of *S. aureus* VAP incidence for all observational and control groups for studies of non-antibiotics based methods (open circle) control groups from studies of topical anti-septics (open blue triangles) and topical antibiotics (SDD) studies (solid red triangles) by year of study publication. Note that the *y*-axis is a logit scale. The linear regression derived from the observational study groups was non-significant (*p* = 0.34) and hence a locally weighted regression and smoothing scatterplot (LOWESS) regression line is given.

There was significant disparity in the summary incidences of both VAP (Figure 2) and *S. aureus* VAP (Figure 3) among the control groups versus the respective benchmarks (Table 1). For VAP and *S. aureus* VAP, the incidences among the control groups in studies of topical antibiotics were each higher by >50% versus the respective benchmarks whereas these incidences for control groups of studies of non-antibiotic based methods were each similar to the corresponding benchmarks. (Table 1). There was no apparent relationship between *S. aureus* VAP and year of publication (Figure 4). Also, there is no impression that the high summary VAP or *S. aureus* VAP incidences was driven by a minority of outlier studies that may have been subject to outbreaks. 

These meta-regression models were each repeated with the following sensitivity tests. Firstly, repeating the models limited to studies obtained from systematic reviews revealed similar findings (Table 2, footnote c and d). Second, as a test for potentially missing studies of topical antibiotics, the models were repeated with component groups of 14 studies of antiseptic based methods [139,140,141,142,143] arbitrarily reclassified as belonging to studies of topical antibiotics. In these models, the coefficients for the control groups of this augmented category of topical antibiotics studies remained significant in both the VAP and *S. aureus* VAP models (Table 2, footnote e).

### 3.4. MRSA-VAP

**Figure 5:**
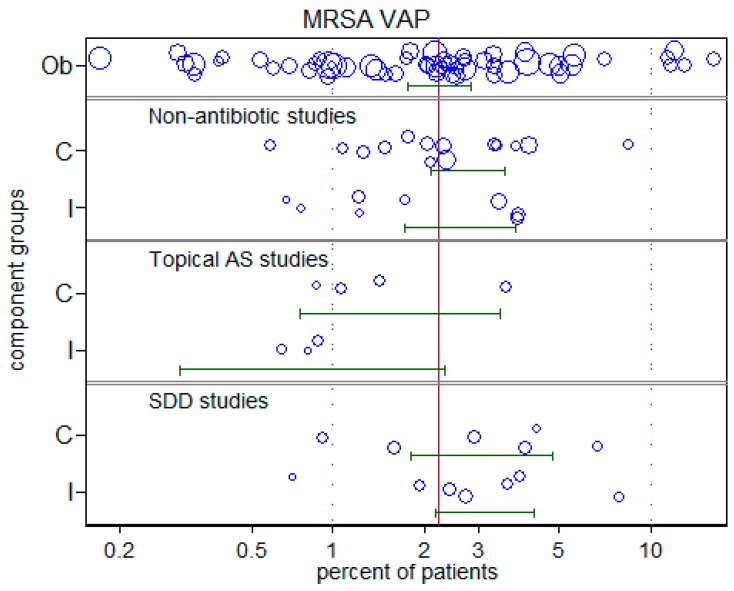
Incidence of methicillin resistant *S. aureus* (MRSA) *S. aureus* VAP. The component (C = control; I = intervention) groups of studies in which there was no intervention (Ob = observational), studies of non-antibiotic methods, topical antiseptics (AS) or topical antibiotics (SDD). (These data are listed in Table S1–S4 (see Additional file 1). The symbol sizes are proportional to the inverse of the study variance. Note that the horizontal axis is a logit scale.

Of the studies analyzed here, the incidence of MRSA *S. aureus* VAP was available from only 108 groups. These data are presented in Figure 5.

### 3.5. Overall and S. aureus bacteremia

**Figure 6:**
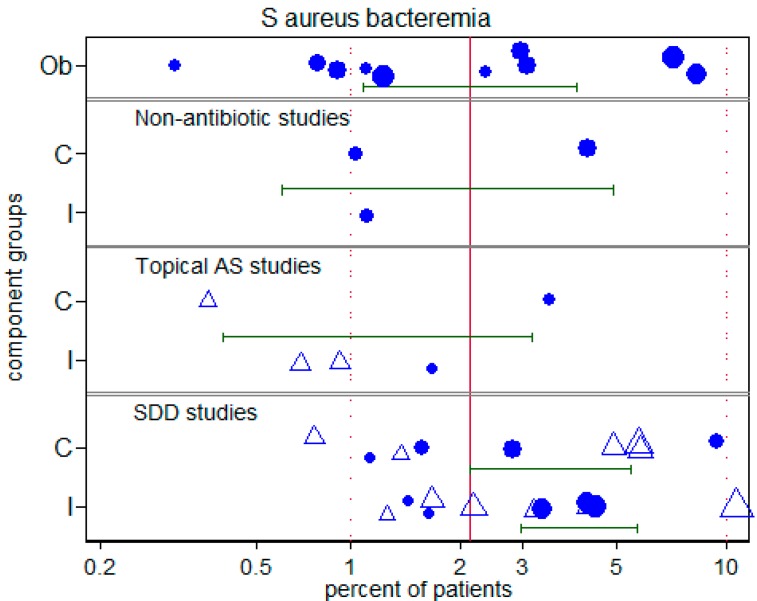
Incidence of *S. aureus* bacteremia. The component (C = control; I = intervention) groups of studies in which there was no intervention (Ob = observational) or studies of non-antibiotic based methods, topical antiseptics (AS) or topical antibiotics (SDD). The *S. aureus* bacteremia benchmark is the summary mean (central vertical line) derived from the observation studies (These data are listed in Table S4 (see Additional file 1). Symbols indicates component groups that came studies that were (open triangles) or were not (solid triangles) from studies in which topical placebo was used to achieve observer blinding. The symbol sizes are proportional to the inverse of the study variance. Note that the horizontal axis is a logit scale.

The incidence of *S. aureus* bacteremia (Table 3) was available from only 38 groups from 21 studies. The *S. aureus* bacteremia incidence was less than 4% for 15 of 18 groups from either observational studies or studies of anti-septic methods. By contrast, the *S. aureus* bacteremia incidence was less than 4% for only 9 of 20 groups from studies of SDD (Figure 6; *p* = 0.014, Chi-square, 1 d.f.).

## 4. Discussion

This is an analysis of the incidences of *S. aureus* VAP and related infections within studies of SDD among a broad range of studies of VAP prevention methods. This analysis is informed by data from other studies in this patient group; *S. aureus* VAP and VAP benchmarks are derived from observational studies without an intervention and studies of non-antibiotic and anti-septic intervention methods provide additional points of reference.

The mean incidence of VAP overall within the control groups of SDD studies is higher than literature derived benchmarks whereas paradoxically the mean incidence in the SDD intervention groups is more similar to the benchmarks. This specific finding is entirely consistent with the apparent >50% reduction in VAP incidence by the SDD intervention as noted in multiple systematic reviews [198,199,200,201,202]. Furthermore, the mean incidence of *S. aureus* VAP and *S. aureus* bacteremia within the control groups of SDD studies are higher than literature derived benchmarks.

There are four key limitations to this analysis, the first being that the studies have been published over a period of three decades. Although there was no apparent trend in *S. aureus* VAP over this time (Figure 4), there was considerable heterogeneity in the interventions, populations, prevalence of antibiotic resistance, and study designs among the studies here. Moreover, the inclusion criteria for all intervention studies here have been intentionally broadly specified. Note that the literature search and analysis has been undertaken by a single author and the analysis is not intended to be a systematic review. The data from each of the studies is displayed in tables and figures to facilitate verification. 

The second limitation is that VAP, and consequently *S. aureus* VAP, is a somewhat subjective end point. Moreover, only a limited number of key group level factors were entered into the regression models and there was no ability to adjust for the underlying patient level risk within the analysis. Hence, the nature of the contextual factor remains unidentified. However, as a counter to these limitations, the findings of a sensitivity analysis limited to studies that used topical placebo to achieve observer blinding gave similar VAP and *S. aureus* VAP incidence estimates. 

The significance of the finding among studies that were placebo controlled is of interest for two reasons. Firstly, the use of a placebo to achieve observer blinding through the concealment of group allocation in controlled trials is a generally accepted marker of higher quality in study design. This is particularly important for study endpoints which lack diagnostic criteria that are objective and unambiguous, such as VAP. Second, the use of topical placebo may serve as a vehicle for cross infection in the ICU setting [224].

The third limitation is that only those studies with data available were able to be included in this analysis. Several studies that included ICU patients not limited to those receiving prolonged mechanical ventilation were not included in the analysis here. For example, in one large Dutch study of SDD [226] only 45 of 10,993 (0.45%) patients had *S. aureus* bacteremia of which only 2 were MRSA. A second is a large American controlled trial of chlorhexidine bathing [227] in which 31 of 9340 patients had *S. aureus* bacteremia (0.3%). A third is a large American controlled trial of targeted chlorhexidine decontamination [228] in which 2007 of 122,646 patients had *S. aureus* bacteremia (1.6%). All three of these studies lack *S. aureus* VAP data, and the proportion of patients that received mechanical ventilation is either 50% or not stated.

The fourth limitation is that the analysis here is inherently observational. Inferences for what may account for the disparate observations can only be speculative. In this regard, a major related limitation is that a contextual effect is difficult to measure reliably. The analysis here has merely identified a high incidence of *S. aureus* VAP and *S. aureus* bacteraemia among the control groups of topical antibiotics studies that remains without an explanation. It remains possible that a high *S. aureus* VAP, VAP or *S. aureus* bacteraemia incidence, served as a prompt to undertake a study of a topical antibiotics regimen. However, this was not explicitly stated in any of the studies analyzed here nor is there any impression of high incidence studies that were outlier as a consequence of having been subject to outbreaks of *S. aureus* VAP. 

The *S. aureus* bacteremia benchmark is derived from only 55 *S. aureus* bacteremia events among 3057 patients among ten observational studies with available data. However, this incidence is comparable with estimates reported in the literature. For example, 45 and 94 *S. aureus* bacteremia events were observed among 4473 and 4913 ICU patients giving incidences of 1.0% [229] and 1.9% [230], respectively. 

The *S. aureus* VAP benchmark and the MRSA-VAP benchmark, being 4.8% (95% CI; 4.2–5.6) and 2.2% (95% CI; 1.7–2.7), respectively, are somewhat more secure and each are also comparable to incidence estimates reported in the literature. Among 1873 mechanical ventilation patients of 56 ICUs of a survey across four multinational regions, there were 65 *S. aureus* VAP (27 MRSA) events observed for an incidence of 3.5% (and 1.5% for MRSA) [54]. 

Are the findings here robust to possible publication bias and undiscovered data? As a counter to this limitation, the findings of a sensitivity analysis using the 14 studies of antiseptic based methods to augment the category of studies of topical antibiotics failed to annul the statistical significance of membership of a control group receiving topical placebo within this augmented category of studies of topical antibiotics. Hence, within the category of the studies of topical antibiotics, there would need to be more that 14 studies with control group incidences of *S. aureus* VAP similar to those among the studies of antiseptic based methods to have been overlooked or unpublished to account for these findings within the meta-regression. 

The disparity in the incidence of *S. aureus* VAP among studies of topical antibiotics versus the respective benchmarks recapitulates similar observations for various endpoints among studies of SDD versus externally derived benchmarks from populations of patients receiving prolonged mechanical ventilation. For example, with respect to *Candida* as a respiratory tract isolate [221], candidemia [222], and overall bacteremia [220] in each case, the incidence of these end points are higher among control groups of studies of SDD versus the respective literature derived benchmarks.

The first study of SDD asked whether the traditional randomized controlled trial format is an appropriate study design for the assessment of decontamination within the context of an ICU [231]. Without the ability to measure and control for the contextual effects resulting from a decontamination intervention, the attribution of any apparent effect to control or to intervention groups will be ambiguous in any single study. Moreover, the use of topical placebo to achieve observer blinding in this context might act as a perfidious confounder [224]. The contextual effects of using topical antibiotics in an attempt to prevent infections in mechanically ventilated patients is a hazard that warrants careful consideration.

## 5. Conclusions

There is an excess in the incidence of *S. aureus* VAP in the studies of topical antibiotics versus benchmarks derived from the observational groups and also versus both the studies of non-antibiotic based methods and the studies of anti-septic methods. This excess is inapparent in any single study of topical antibiotics examined in isolation and is apparent only on reference to an external benchmark incidence. This excess cannot be readily accounted for. It likely represents herd peril resulting from the use of SDD. This together with the excess of *S. aureus* bacteremias, and VAP overall implies a profound contextual effect within the topical antibiotics studies.

## Figures and Tables

**Table 1:** Characteristics of studies.

Variable	Observational (No Intervention)	Non-Antibiotic Studies	Topical Antiseptic Studies	Topical Antibiotic Studies
Supplemental material	Table S1	Table S2	Table S3	Table S4
Number of studies ^a^	106	50	14	26
Publication year (range)	1987–2016	1987–2016	2000–2014	1987–2015
Studies sourced from systematic reviews ^b^	45	32	8	24
Topical placebo used in study	NA	8	7	14
Bronchoscopic sampling for VAP diagnosis (n) ^c^	58	22	5	8
Trauma ICU’s (n) ^d^	21	11	3	8
North American ICU’s (n) ^e^	21	13	2	1
Patients per study group median (IQR) ^f^	279 135–618	75 61–149	114 52–147	52 36–126
VAP incidence per 100 patients				
Observational (mean) 95% CI n	20.8 19.0–22.8 115			
Control (mean) 95% CI n		21.6 19.2–24.1 50	20.1 13.6–28.9 14	32.7 26.9–38.9 27
Intervention (mean) 95% CI n		16.5 14.2–19.2 43	13.8 8.6–21.4 14	14.8 12.0–18.1 26
*S. aureus* VAP incidence per 100 patients				
Observational (mean) 95% CI n	4.8 4.2–5.6 115			
Control (mean) 95% CI n		7.2 6.3–8.3 50	5.1 2.7–9.3 14	9.6 6.9–13.2 27
Intervention (mean) 95% CI n		5.3 4.1–6.6 43	3.1 1.6–6.1 14	6.4 4.8–8.3 26

^a^ Note, several studies had more than one control and or intervention group. Hence the number of groups does not equal the number of studies; ^b^ Studies that were sourced from 19 systematic reviews (200–204, 207–220); ^c^ Bronchoscopic versus tracheal sampling for VAP diagnosis; ^d^ Trauma ICU arbitrarily defined as an ICU with more than 50% of admissions for trauma; ^e^ Number of studies originating from either the United States of America or Canada; ^f^ Data is median and inter-quartile range (IQR).

**Table 2:** Logit regression models ^a^.

		VAP			*S. aureus* VAP	
Factor	Coefficient ^b^	95% CI	*p*	Coefficient ^b^	95% CI	*p*
Groups from observational studies (reference group)	−1.30	−1.62 to −0.97		−2.70	−3.01 to −2.29	
Control groups						
Non- antibiotic studies, no placebo	+0.04	−0.21 to +0.29	0.77	+0.10	−0.15 to +0.33	0.42
Non- antibiotic studies; with topical placebo	−0.08	−0.60 to +0.45	0.77	+0.33	−0.14 to +0.81	0.17
Topical antiseptic studies; no placebo	−0.11	−0.66 to +0.44	0.69	+0.01	−0.55 to +0.57	0.97
Topical antiseptic studies; with topical placebo	−0.20	−0.81 to +0.41	0.53	+0.07	−0.48 to +0.62	0.81
Topical antibiotic studies; no placebo	+0.50 ^c^	+0.05 to +0.95	0.03	+0.31 ^d,e^	−0.08 to +0.69	0.12
Topical antibiotic studies; with topical placebo	+0.38 ^c^	−0.04 to +0.79	0.08	+0.48 ^d,e^	+0.15 to +0.82	0.004
Intervention groups						
Non-antibiotic studies	−0.34	−0.60 to −0.09	0.008	−0.14	−0.39 to +0.12	0.30
Topical antiseptic studies;	−0.77	−1.18 to −0.35	0.001	−0.53	−1.03 to −0.03	0.039
Topical antibiotic studies;	−0.57	−0.90 to −0.23	0.001	−0.21	−0.53 to +0.11	0.20
Trauma ICU ^f^	+0.39	+0.18 to +0.59	0.001	+0.86	+0.64 to +1.08	0.001
Mode of diagnosis ^g^	−0.03	−0.21 to +0.14	0.73	+0.08	−0.11 to +0.28	0.41
North American study ^h^	−0.31	−0.56 to −0.06	0.01	−0.28	−0.54 to −0.02	0.04
Year of publication ^i^	+0.01	−0.01 to +0.01	0.88	−0.01	−0.03 to +0.001	0.08

^a^ Abbreviations; ICU, Intensive care unit; VAP, ventilator associated pneumonia; ^b^ Interpretation. For each model the reference group is the observational study (benchmark) groups and this coefficient equals the difference in logits from 0 (a logit equal to 0 equates to a proportion of 50%; a logit equal to −1.4 equates to a proportion of 20%; a logit equal to −3 equates to a proportion of 4.8%). The other coefficients represent the difference in logits for groups positive for that factor versus the reference group; ^c^ The VAP logit regression model was repeated limited to studies that had been cited in one of the systematic reviews. In this model the coefficients were +0.47; +0.01 to +0.93, *p* = 0.045 (without topical placebo use) and +0.37; −0.07 to +0.81, *p* = 0.097 (with topical placebo use); ^d^ The *S. aureus* VAP logit regression model was repeated limited to studies that had been cited in one of the systematic reviews. In this model the coefficients were +0.45; −0.10 to +1.00, *p* = 0.11 (without topical placebo use) and +0.59; +0.08 to +1.09, *p* = 0.023 (with topical placebo use); ^e^ The *S. aureus* VAP logit regression model was repeated with component groups of Topical antiseptic studies arbitrarily reclassified as belonging to studies of topical antibiotics. In this collapsed model the coefficients were +0.28; −0.11 to +0.67, *p* = 0.16 (without topical placebo use) and +0.45; +0.06 to +0.84, *p* = 0.02 (with topical placebo use); ^f^ Trauma ICU arbitrarily defined as an ICU for which >50% of admissions were for trauma; ^g^ Diagnosis of VAP using bronchoscopic versus tracheal based sampling; ^h^ Originating from an ICU in The United States of America or Canada; ^i^ Year of study publication with the coefficient representing the increment for each year post 1985.

**Table 3:** *S. aureus* bacteremia data.

		*S. aureus* bacteremia Incidence Proportion	
Factor	Per 100 Patients	95% CI	Number of Groups
Groups from observational studies	2.1	1.1–4.1	10
Non-antibiotic studies			
Control and Intervention groups	1.8	0.6–5.1	3
Topical antiseptic studies;			
Control and Intervention groups	1.2	0.4–3.1	5
Topical antibiotic studies;			
Control groups	3.8	2.1–5.7	9
Intervention groups	4.2	2.9–5.9	11

## Supplementary Materials

The following are available online at www.mdpi.com/2076-2607/6/1/2/s1.

Supplementary File 1
