# Supplementary material for: Unusually High Incidences of Staphylococcus aureus Infection within Studies of Ventilator Associated Pneumonia Prevention Using Topical Antibiotics: Benchmarking the Evidence Base
Source: Microorganisms. 2018 Jan 4;6(1):2. doi: 10.3390/microorganisms6010002 (PMC5874616; doi:10.3390/microorganisms6010002)
Supplement: Supplementary File 1 [file microorganisms-06-00002-s001.pdf]

Additional file contents:

Table S1: *S. aureus* VAP data: observational studies (Benchmark groups) 2 - 5
Table S2: *S. aureus* VAP data: non-antibiotic-based methods of VAP prevention 6 - 10
Table S3: *S. aureus* VAP data: studies of topical antiseptic based methods of VAP prevention 11 – 12
Table S4: *S. aureus* VAP data: studies of SDD 13 – 15
Table S5: *S. aureus* bacteremia data: all studies 16
References 18 - 29

Table S1: *S. aureus* VAP data: observational studies (Benchmark groups)

Author	Year	Ref	Notes [a]	MVD	Patients (n)	VAP (n)	VAP %	S aureus (n)	S aureus %	MRSA (n)
**A'court**	1993	1	Tr	9	150	33	22.0	4	2.7	
**Alvarez-Lerma**	1996	2		9.2	6494	519	8.0	102	1.6	
**Antonelli**	1994	3	B, Tr	11.9	124	41	33.1	10	8.1	
**Apostolopoulou**	2003	4	Sr	12.6	175	56	32.0	9	5.1	
**Baker**	1996	5	US, B, Tr, Sr	999	514	30	5.8	5	1.0	
**Bekaert**	2011	6		8	4479	685	15.3	133	3.0	49
**Bercault_IHT**	2005	7		9	118	31	26.3	2	1.7	
**Bercault_noINT**	2005	7	I	11	118	12	10.2	2	1.7	
**Berrouane_all**	1998	8	B, Tr	11	565	129	22.8	71	12.6	14
**Bochicchio**	2004	9	US, Tr, Sr	10.5	678	125	18.4	50	7.4	9
**Bonten'94**	1994	10	B, Sr		64	11	17.2	3	4.7	
**Boots**	2008	11	B	10.6	412	58	14.1	32	7.8	17
**Bornstain**	2004	12	B	8.5	747	80	10.7	17	2.3	6
**Braun**	1986	13	US, Tr, Sr	6	66	15	22.7	6	9.1	
**Bregeon**	1997	14	B	10.3	660	223	33.8	34	5.2	
**Bronchard**	2004	15	B, Tr, Sr	17	109	45	41.3	26	23.9	1
**Cade**	1993	16		4	98	35	35.7	13	13.3	10
**Cavalcanti**	2006	17	B, Tr	5	190	62	32.6	18	9.5	
**Cenderero**	1999	18	B	6.5	123	19	15.4	9	7.3	3
**Chaari**	2015	19	Tr	8.2	175	48	27.4	6	3.4	
**Chastre**	1998	20	B, Sr	19	243	84	34.6	18	7.4	
**Chevret**	1993	21		5	255	55	21.6	23	9.0	
**Cook_non-trauma**	2010	22		5.5	2080	70	3.4	14	0.7	12
**Cook_trauma**	2010	22	Tr, US	7.3	511	91	17.8	15	2.9	10
**Craven-medical**	1988	23	US	4	277	47	17.0	9	3.2	
**Craven-surgical**	1988	23	US	3.6	521	49	9.4	12	2.3	
**Daschner**	1988	24	Sr	6	116	36	31.0	13	11.2	
**de_Latore**	1995	25	B, Sr	10.2	80	12	15.0	3	3.8	
**Ertugrul**	2006	26	Tr	9.7	100	28	28.0	12	12.0	11
**Evans**	2010	27	US, Tr	8	416	101	24.3	40	9.6	22
**Ewig**	1999	28	B, Tr	6	48	10	20.8	5	10.4	0
**Fagon'89**	1989	29	B, Sr	13	567	49	8.6	17	3.0	
**Gacouin**	2009	30	B	11	361	76	21.1	21	5.8	9
**Garrouste-Orgas**	1997	31	B	11	86	31	36.0	13	15.1	13
**George**	1998	32	US	6	223	28	12.6	8	3.6	4
**Georges**	2000	33	B, Sr	18	135	35	25.9	11	8.1	
**Giard**	2008	34	B	8	7236	946	13.1	193	2.7	
**Gruson-95-96**	2000	35	B	11.6	1004	231	23.0	67	6.7	40
**Gruson-97-98**	2000	35	B	11.9	1029	161	15.6	54	5.2	20
**Gruson-99-01**	2003	36	B	11.6	823	134	16.3	26	3.2	

Table S1 (continued): *S. aureus* VAP data: observational studies (Benchmark groups)

Author	Year	Ref	Notes [a]	MV D	Patients (n)	VAP (n)	VAP %	S aureus (n)	S aureus %	MRSA (n)
**Guérin**	1997	37	B	8.9	260	27	10.4	3	1.2	1
**Guimaraes**	2006	38	Sr	7	278	106	38.1	7	2.5	7
**Gursel**	2010	39		10	92	59	64.1	13	14.1	12
**Heyland**	1999	40	US, B	16	1014	177	17.5	64	6.3	4
**Hugonnet**	2007	41	B	4.8	936	209	22.3	55	5.9	
**Hyllienmark**	2007	42	B	5	221	33	14.9	2	0.9	0
**Ibáñez**	2000	43		7.6	30	6	20.0	3	10.0	
**Ibrahim'00**	2000	44	US, Sr	4.7	1882	397	21.1	143	7.6	81
**Jacobs**	1990	45	Sr		24	13	54.2	2	8.3	
**Jaillette**	2011	46		15	439	137	31.2	22	5.0	13
**Jaimes**	2007	47	Sr	9.6	270	60	22.2	10	3.7	
**Jimenez**	1989	48	Sr	5.6	77	18	23.4	2	2.6	
**Kallel**	2005	49	Tr	13.7	241	77	32.0	15	6.2	2
**Kanafani**	2003	50	Sr	17	70	33	47.1	3	4.3	0
**Kollef' 93**	1993	51	US, Sr	7.4	277	43	15.5	9	3.2	
**Kollef '95**	1995	52	US, Sr		314	87	27.7	17	5.4	8
**Kollef '97**	1997	53	US, Sr	5.4	521	77	14.8	25	4.8	12
**Kollef'14_All**	2014	54	US	11.4	1873	293	15.6	65	3.5	27
**Koss– N**	2001	55	US	10.3	87	17	19.5	3	3.4	
**Koss– P**	2001	55	I	7.5	66	24	36.4	4	6.1	
**Kunac**	2014	56	US, B, Tr	5	716	206	28.8	62	8.7	40
**Lepelletier**	2010	57	T, Tr	13	161	34	21.1	34	21.1	5
**Luna**	2003	58	B	7.6	427	63	14.8	19	4.4	13
**Luyt**	2005	59		35	290	69	23.8	12	4.1	4
**Magnason**	2008	60			280	21	7.5	1	0.4	
**Magret_non-trauma**	2010	61		5	2082	337	16.2	84	4.0	42
**Magret_trauma**	2010	61	Tr	5	354	128	36.2	32	9.0	10
**Mahul**	1992	62	B, Sr	16.6	145	30	20.7	10	6.9	
**Makris**	2011	63	I, B	20	152	44	28.9	3	2.0	
**Markowicz**	2000	64	B, Sr	11.7	744	162	21.8	74	9.9	40
**Memish**	2000	65	Sr	8	202	41	20.3	16	7.9	
**Michel**	2005	66	B		299	41	13.7	12	4.0	1
**Moine**	2002	67	B, Sr	9.7	764	89	11.6	19	2.5	14
**Myny**	2005	68	Sr	6	385	89	23.1	27	7.0	7
**Nguile-Makao**	2010	69	B	7	2873	434	15.1	89	3.1	
**Nielsen**	1992	70		3.8	242	23	9.5	5	2.1	
**Noor**	2005	71	Sr	6	250	70	28.0	11	4.4	7
**Nseir**	2005	72	Sr	24	1241	77	6.2	15	1.2	8

Table S1 (continued): *S. aureus* VAP data: observational studies (Benchmark groups)

Author	Year	Ref	Notes [a]		MVD	Patients (n)	VAP (n)	VAP %	S aureus (n)	S aureus %	MRSA (n)
**Papazian**	1996	73	B, Sr		16	586	97	16.6	20	3.4	
**Potgieter**	1987	74	Sr		9.2	250	51	20.4	23	9.2	6
**Raineri**	2010	75	I, B	B	4.4	822	44	5.4	11	1.3	4
**Raineri**	2010	75	B	B	5.6	827	68	8.2	22	2.7	3
**Ramirez**	2016	76	B	B	7	440	71	16.1	8	1.8	
**Rello'91**	1991	77	B, Sr	B	7.9	264	58	22.0	15	5.7	
**Rello'92**	1992	78	B	B		208	53	25.5	22	10.6	0
**Rello'96**	1996	79	B, Sr	B	8	83	21	25.3	0.5	0.0	
**Rello'02**	2002	80	US, Sr		5.4	9080	842	9.3	143	1.6	
**Rello'03**	2003	81	Sr	T	7	99	18	18.2	2	2.0	1
**Resende**	2013	82		T	24	126	33	26.2	5	4.0	
**Reusser**	1989	83	Sr	T	7	40	15	37.5	6	15.0	
**Rezai**	2017	84		T		562	205	36.5	24	4.3	
**Rincón-Ferrari**	2004	85	B, Tr	B	10	310	72	23.2	27	8.7	
**Rodrigues**	2009	86		T	10	233	64	27.5	11	4.7	6
**Rodriguez**	1991	87	US, Tr, Sr	T	11	294	130	44.2	37	12.6	14
**Ruiz-Santana**	1987	88	Sr	B	7	1005	180	17.9	12	1.2	8
**Salahuddin**	2004	89	I	T		333	43	12.9	3	0.9	
**Salahuddin**	2004	89		T		344	19	5.5	3	0.9	
**Salata**	1987	90	US, B, Sr	B	13.5	51	21	41.2	2	3.9	
**Shahin**	2013	91	US	T	6	267	29	10.9	6	2.2	
**Sofianou**	2000	92	Sr	T	8	198	67	33.8	13	6.6	13
**Stéphan**	2006	93	Tr, Sr	T	5	175	78	44.6	43	24.6	2
**Tan**	2016	94		T	9	618	286	46.3	23	3.7	
**Tejada-Artigas**	2001	95	B, Tr, Sr	B	8	103	23	22.3	11	10.7	
**Timsit**	1996	96	B, Sr	B	12	387	56	14.5	18	4.7	
**Torres**	1990	97	B, Sr	B	4	322	78	24.2	2	0.6	
**Trouillet**	1998	98	B	B	17	498	135	27.1	52	10.4	32
**Urli**	2002	99		T		178	116	65.2	40	22.5	20
**Valles**	2007	100	Sr	T	12	60	40	66.7	9	15.0	0
**Vanhems**	2011	101	B			3387	367	10.8	137	4.0	
**Verhamme**	2007	102			7.7	4000	298	7.4	56	1.4	7
**Violan**	1998	103	B, Sr	B	13	314	82	26.1	26	8.3	1
**Woske**	2001	104	B, Sr	B	15	103	49	47.6	29	28.2	1
**Xie**	2011	105			2.5	4155	868	20.9	92	2.2	41
**Zahar**	2009	106	B		8.84	1233	208	16.9	51	4.1	

Table S1 footnotes

Tr – Data originating from a study for which the majority of ICU admission were for trauma

US - Data originating from a study based in the United States of America or Canada

B – VAP diagnosis based on bronchoscopic based methods for sampling.

Sr - These studies were cited in the following source systematic reviews.

- Agrafiotis M, Siempos II, Ntaidou TK, Falagas ME. Attributable mortality of ventilator-associated pneumonia: a meta-analysis. The International Journal of Tuberculosis and Lung Disease. 2011 Sep 1;15(9):1154-63.
- Melsen WG, Rovers MM, Bonten MJM: Ventilator-associated pneumonia and mortality: A systematic review of observational studies. *Crit Care Med* 2009, 37:2709–2718.
- Safdar N, Dezfulian C, Collard HR, Saint S: Clinical and economic consequences of ventilator-associated pneumonia: a systematic review. *Crit Care Med* 2005, 33:2184–93.

Table S2: *S. aureus* VAP data: non-antibiotic-based methods of VAP prevention [a]

Author	Year	Ref	Notes [b]	MVD	Patients (n)	VAP (n)	VAP %	S aureus (n)	S aureus %	MRSA (n)
**control groups**										
**Acosta-escribano**	2010	107	Tr, Sr	8.9	54	31	57.4	4	7.4	8.9
**Bonten '95**	1995	108	B, Pl, Sr	9.2	74	16	21.6	7	9.5	9.2
**Boots'06_All**	2006	109	Sr	13.2	381	59	15.5	28	7.3	13.2
**Combes**	2000	110	Tr, Sr	11.3	50	4	8.0	4	8.0	11.3
**Cook**	1998	111	US, Pl, Sr	7	596	114	19.1	44	7.4	7
**Daumal**	1999	112			174	25	14.4	7	4.0	
**Djedaini**	1995	113	B		61	6	9.8	0.5	0.0	
**Drakulovic**	1999	114	Sr	7.8	47	11	23.4	4	8.5	7.8
**Dreyfuss**	1991	115	B, Sr	12.8	35	11	31.4	2	5.7	12.8
**Dreyfuss**	1995	116	B, Sr	12.5	70	8	11.4	2	2.9	12.5
**Driks**	1987	117	Sr		69	16	23.2	4	5.8	
**Fabian_all**	1993	118	US, Tr, Sr	6	278	81	29.1	32	11.5	6
**Forestier**	2008	119	B, Pl, Tr, Sr		106	21	19.8	11	10.4	
**Heyland**	1999	120	US, Pl	7.8	46	7	15.2	0.5	0.0	7.8
**Holzapfel_C_93**	1993	121	B, Sr	9.5	149	17	11.4	8	5.4	9.5
**Holzapfel_C_99**	1999	122	B	14	200	51	25.5	21	10.5	14
**Kappstein**	1991	123	Tr		55	25	45.5	9	16.4	
**Kirschenbaum**	2002	124	US, Sr	12	20	10	50.0	1	5.0	12
**Kirton**	1997	125	US, Tr, Sr	16.3	140	22	15.7	6	4.3	16.3
**Knight**	2009	126	Pl, Sr	5	129	17	13.2	1	0.8	5
**Kollef '95**	1995	127	US, Sr	15	300	80	26.7	22	7.3	15
**Kollef '97**	1997	128	US, Sr	5.6	521	77	14.8	42	8.1	5.6
**Kollef '98**	1998	129	US, Sr	3.7	147	15	10.2	7	4.8	3.7
**Kostadima**	2005	130			21	8	38.1	3	14.3	
**Lacherade '05**	2005	131	B, Sr	14.9	184	53	28.8	16	8.7	14.9
**Lacherade '10**	2010	132	B, Sr	8	164	42	25.6	8	4.9	8
**Laueny**	2014	133	Tr	10	91	11	12.1	6	6.6	10
**Lorente '03**	2003	134	B	14.8	116	26	22.4	8	6.9	14.8
**Lorente '04**	2004	135	B, Sr	16	143	33	23.1	6	4.2	16
**Lorente'05**	2005	136	B, Sr	12.7	233	42	18.0	11	4.7	12.7
**Lorente'06**	2006	137	B, Sr	9.5	221	31	14.0	8	3.6	9.5
**Lorente'06**	2006	138	B, Sr	20.8	51	8	15.7	5	9.8	20.8
**Lorente'07**	2007	139	Sr	11.1	140	31	22.1	8	5.7	11.1
**Lorente'14**	2014	140		11.1	150	33	22.0	5	3.3	11.1

Table S2 (continued): *S. aureus* VAP data: non-antibiotic-based methods of VAP prevention [a]

Author	Year	Ref	Notes [b]	MVD	Patients (n)	VAP (n)	VAP %	S aureus (n)	S aureus %	MRSA (n)
**control groups**										
**Manzano**	2008	141			63	16	25.4	9	14.3	2
**Martin**	1993	142	US, Pl	4	66	6	9.1	1	1.5	
**Morrow**	2010	143	US, B, Pl, Sr	9.6	73	28	38.4	14	19.2	
**Nseir**	2011	144	B	8	61	16	26.2	3	4.9	
**Pickworth**	1993	145	US, Tr, Sr	2.9	44	5	11.4	1	2.3	
**Pneumatikos**	2006	146	Pl, Tr	15	40	11	27.5	4	10.0	
**Prod'hom_A**	1994	147	Sr	6	81	18	22.2	5	6.2	
**Reigneir**	2013	148	B	7	222	35	15.8	17	7.7	
**Rumbak**	2004	149	US		60	15	25.0	5	8.3	
**Ryan_C**	1993	150	US, Sr	5.1	56	7	12.5	1	1.8	
**Smulders**	2002	151	Sr	7.1	75	12	16.0	3	4.0	
**Staudinger**	2010	152	B, Sr	14	75	17	22.7	2	2.7	
**Thomachot**	1998	153	B	12	66	21	31.8	7	10.6	
**Thomachot**	1999	154	Tr	11.1	77	24	31.2	8	10.4	
**Thomachot**	2002	155	Tr	9.5	84	22	26.2	7	8.3	
**Valencia**	2007	156	B, Sr	6	69	10	14.5	2	2.9	0
**Zeng**	2016	157		17	117	59	50.4	16	13.7	

Table S2: *S. aureus* VAP data: non-antibiotic-based methods of VAP prevention [a]

Author	Year	Ref	Notes [b]	MVD	Patients (n)	VAP (n)	VAP %	S aureus (n)	S aureus %	MRSA (n)
**intervention groups**										
**Acosta-escribano**	2010	107	Tr, Sr	7.3	50	16	32.0	8	16.0	
**Bonten '95**	1995	108	B, Sr	9.2	67	15	22.4	4	6.0	
**Cook**	1998	111	US, Sr	8	604	98	16.2	36	6.0	
**Daumal**	1999	112			187	30	16.0	9	4.8	
**Djedaini**	1995	113			68	8	11.8	2	2.9	
**Drakulovic**	1999	114	Sr	9.5	39	2	5.1	0.5	0.0	0
**Dreyfuss**	1995	115	B, Sr	10	61	6	9.8	0.5	0.0	
**Dreyfuss**	1991	116	B, Sr	10	28	8	28.6	1	3.6	
**Driks**	1987	117	Sr		61	7	11.5	0.5	0.0	
**Forestier**	2008	119	B, Tr, Sr		102	19	18.6	12	11.8	
**Heyland**	1999	120	US	8.5	49	3	6.1	1	2.0	
**Holzapfel_I_99**	1999	121	B	16	199	37	18.6	7	3.5	
**Kantorova All**	2004	158	Tr, Sr		287	25	8.7	5	1.7	
**Kappstein**	1991	123	Tr		49	12	24.5	11	22.4	
**Kirton**	1997	125	US, Tr, Sr	20.4	140	9	6.4	6	4.3	
**Knight**	2009	126		5	130	12	9.2	0.5	0.0	
**Lacherade '05**	2005	131	B, Sr	13.5	185	47	25.4	18	9.7	6
**Lacherade '10**	2010	132	B, Sr	7	169	25	14.8	2	1.2	0
**Laueny**	2014	133	Tr	15	98	37	37.8	17	17.3	0
**Lorente '03**	2003	134	B, Sr	13.7	114	29	25.4	7	6.1	
**Lorente '04**	2004	135	B, Sr	20	161	37	23.0	14	8.7	6
**Lorente'05**	2005	136	B, Sr	12.1	210	43	20.5	10	4.8	8
**Lorente'06**	2006	137	B, Sr	9.9	236	33	14.0	8	3.4	3
**Lorente'06**	2006	138	B, Sr	19.5	53	21	39.6	2	3.8	0
**Lorente'07**	2007	139	Sr	10.5	140	11	7.9	2	1.4	1
**Lorente'14**	2014	140		10.5	134	15	11.2	1	0.7	1
**Manzano**	2008	141			64	6	9.4	4	6.3	1
**Martin**	1993	142	US	4	65	2	3.1	0.5	0.0	
**Morrow**	2010	143	US, B, Sr	9.5	73	13	17.8	8	11.0	
**Nseir**	2011	144	B	8	61	6	9.8	1	1.6	

Table S2: *S. aureus* VAP data: non-antibiotic-based methods of VAP prevention [a]

Author	Year	Ref	Notes [b]	MVD	Patients (n)	VAP (n)	VAP %	S aureus (n)	S aureus %	MRSA (n)
**intervention groups**										
**Pneumatikos**	2006	146	Tr	16	39	6	15.4	2	5.0	
**Prod'hom_R**	1994	147	Sr	6	80	22	27.5	4	5.0	
**Prod'hom_S**	1994	147	Sr	5	83	11	13.3	2	2.4	
**Reigneir**	2013	148	B	7	227	38	16.7	10	4.4	
**Rumbak**	2004	149	US		60	3	5.0	1	1.7	
**Ryan_S**	1993	150	US, Sr	5.6	58	8	13.8	2	3.4	
**Smulders**	2002	151		7.9	75	3	4.0	1	1.3	
**Staudinger**	2010	152	B, Sr	8	75	8	10.7	2	2.7	
**Thomachot**	1998	153	B	12	70	26	37.1	8	11.4	
**Thomachot**	1999	154	Tr	12.3	63	21	33.3	7	11.1	
**Thomachot**	2002	155	Tr	8.6	71	10	14.1	5	7.0	
**Valencia**	2007	156	B, Sr	5	73	11	15.1	2	2.7	1
**Zeng**	2016	157		12	118	43	36.4	12	10.2	

Table S2 footnotes

a. Study interventions; histamine 2 receptor antagonist; proton pump inhibitor; feeding by the gastric route; feeding by the small bowel route; open tracheal suction system; closed tracheal suction system; Heated humidifier; heat and moisture exchanger changed every 24 hours; heat and moisture exchanger changed every 48 hours; subglottic secretion drainage; no circuit changes; circuit changes at 48 hours.

b. Notes

Pl – topical placebo used to achieve observe binding

Tr – Data originating from a study for which the majority of ICU admission were for trauma

US - Data originating from a study based in the United States of America or Canada

B – VAP diagnosis based on bronchoscopic based methods for sampling.

Sr - These studies were cited in the following source systematic reviews.

- Messori A, Trippoli S, Vaiani M, Gorini M, Corrado A: Bleeding and pneumonia in intensive care patients given ranitidine and sucralfate for prevention of stress ulcer: meta-analysis of randomised controlled trials. *BMJ* 2000, 321:1103–1106.
- Huang J, Cao Y, Liao C, Wu L, Gao F: Effect of histamine-2-receptor antagonists versus sucralfate on stress ulcer prophylaxis in mechanically ventilated patients: a meta-analysis of 10 randomized controlled trials. *Crit Care* 2010, 14:R194.
- Alhazzani W, Almasoud A, Jaeschke R, Lo BW, Sindi A, Altayyar S, Fox-Robichaud A: Small bowel feeding and risk of pneumonia in adult critically ill patients: a systematic review and meta-analysis of randomized trials. *Crit Care* 2013, 17:R127.
- Melsen WG, Rovers MM, Bonten MJM: Ventilator-associated pneumonia and mortality: A systematic review of observational studies. *Crit Care Med* 2009, 37:2709–2718.

- Safdar N, Dezfulian C, Collard HR, Saint S: Clinical and economic consequences of ventilator-associated pneumonia: a systematic review. *Crit Care Med* 2005, 33:2184–93.
- Han J, Liu Y. Effect of ventilator circuit changes on ventilator-associated pneumonia: a systematic review and meta-analysis. *Respiratory care*, 2010; 55: 467-474.
- Subirana M, Solà I, Benito S: Closed tracheal suction systems versus open tracheal suction systems for mechanically ventilated adult patients. *Cochrane Database Syst Rev* 2007, 4: CD004581;
- Siempos II, Vardakas KZ, Kopterides P, Falagas ME. Impact of passive humidification on clinical outcomes of mechanically ventilated patients: A meta-analysis of randomized controlled trials. *Crit Care Med* 2007; 35: 2843-51;
- Muscedere J, Rewa O, McKechnie K, Jiang X, Laporta D, Heyland DK. Subglottic secretion drainage for the prevention of ventilator-associated pneumonia: a systematic review and meta-analysis. *Crit Care Med* 2011; 39:1985–1991.
- Delaney A, Gray H, Laupland KB, Zuege DJ. Kinetic bed therapy to prevent nosocomial pneumonia in mechanically ventilated patients: a systematic review and meta-analysis. *Crit Care* 2006; 10:R70;
- Sud S, Friedrich JO, Taccone P, Polli F, Adhikari NK, Latini R, Gattinoni L. Prone ventilation reduces mortality in patients with acute respiratory failure and severe hypoxemia: systematic review and meta-analysis. *Inten Care Med* 2010; 36(4); 585-599.
- Siempos II, Vardakas KZ, Falagas ME. Closed tracheal suction systems for prevention of ventilator-associated pneumonia. *Brit J Anaesthesia*, 2008; 100(3): 299-306.

Table S3: *S. aureus* VAP data: studies of topical antiseptic based methods of VAP prevention [a]

Author	Year	Ref	Notes [b]	MVD	Patients (n)	VAP (n)	VAP %	S aureus (n)	S aureus %	MRSA (n)
**control groups**										
**Cabov**	2010	159	Pl		30	6	20	2	6.7	
**Caruso**	2009	160	B	11	132	31	23.5	5	4	5
**Fourrier'00**	2000	161	Sr	18	30	15	50.0	3	10.0	
**Fourrier'05**	2005	162	Pl, Sr	10.6	114	12	10.5	2	1.8	1
**Genuit (C & T)**	2001	163	US		78	27	34.6	7	9.0	
**Koeman**	2006	164	Pl, Sr	7	130	23	17.7	5	3.8	
**Kollef'08**	2008	165	US	4	743	56	7.5	16	2.2	
**Lorente'12**	2012	166	Sr	9.1	219	24	11.0	4	1.8	3
**Mori H**	2006	167		6	414	25	6.0	5	1.2	4
**Panchachai**	2009	168	Pl, Sr	4	83	15	18.1	3	3.6	
**Seguin – CC**	2006	169	B, Tr, Sr	12	31	13	41.9	7	22.6	0
**Seguin – SC**	2006	169	B, Pl, Tr, Sr	10	31	12	38.7	7	22.6	0
**Seguin**	2014	170	B, Pl, Tr		72	20	27.8	11	15.3	
**Tantipong**	2008	171	Pl, Sr		52	10	19.2	0.5	0.0	
**intervention groups**										
**Cabov**	2010	159			30	1	3.3	0.5	0.0	
**Camus MCh**	2005	178, 200	B, Sr		130	24	18.5	1	0.8	1
**Caruso**	2009	160	B	11	130	14	10.8	1	0.8	
**Fourrier'00**	2000	161	Sr	13	30	5	16.7	0.5	0.0	
**Fourrier'05**	2005	162	Sr	11.7	114	13	11.4	1	0.9	0
**Koeman-Ch**	2006	164	Sr	9.2	127	13	10.2	2	1.6	
**Kollef'08_silverETT**	2008	165	US, B	4	766	37	4.8	9	1.2	
**Lorente'12**	2012	166	Sr	9.7	217	21	9.7	4	1.8	2
**Mori H**	2006	167		5.9	1248	25	2.0	7	0.6	1
**Panchachai**	2009	168	Sr	3	88	14	15.9	2	2.3	
**Pobo**	2009	172	B		73	18	24.7	9	12.3	
**Seguin-PVI**	2006	169	B, Tr, Sr	9	36	3	8.3	3	8.3	0
**Seguin**	2014	170	B, Tr, Sr		78	24	30.8	14	17.9	
**Tantipong**	2008	171	Sr		58	5	8.6	0.5	0.0	

Table S3 footnotes

a. Study interventions; chlorhexidine alone; chlorhexidene with toothbrushing; chlorhexidene with mupirocin; saline installation; sliver endotracheal tubing; povidone-iodeine.

b. Notes

Pl – topical placebo used to achieve observe binding

Tr – Data originating from a study for which the majority of ICU admission were for trauma

US - Data originating from a study based in the United States of America or Canada

B – VAP diagnosis based on bronchoscopic based methods for sampling.

Sr - These studies were cited in the following source systematic reviews.

- Silvestri L, Weir I, Gregori D, Taylor D, Van Saene J, Van Saene H. Effectiveness of oral chlorhexidine on nosocomial pneumonia, causative microorganisms and mortality in critically ill patients: a systematic review and meta-analysis. Minerva Anestesiol. 2014;80(7):805-20.
- Pileggi C, Bianco A, Flotta D, Nobile CG, Pavia M. Prevention of ventilator-associated pneumonia, mortality and all intensive care unit acquired infections by topically applied antimicrobial or antiseptic agents: a meta-analysis of randomized controlled trials in intensive care units. *Crit Care* 2011; 15:R155.
- Chan EY, Ruest A, Meade MO, Cook DJ. Oral decontamination for prevention of pneumonia in mechanically ventilated adults: systematic review and meta-analysis. *BMJ.* 2007; 334:889–900.
- Labeau SO, Van de Vyver K, Brusselaers N, Vogelaers D, Blot SI: Prevention of ventilator-associated pneumonia with oral antiseptics: a systematic review and meta-analysis. *Lancet Infect Dis* 2011, 11:845-854.

Table S4: *S. aureus* VAP data: studies of SDD [a]

Author	Year	Ref	Notes [b]	MV D	Patients (n)	VAP (n)	VAP %	S aureus (n)	S aureus %	MRSA (n)
**control groups**										
**Abele-Horn**	1997	173	Tr, Sr	15	30	20	66.7	5	16.7	
**Aerdts**	1991	174	Sr	23	39	27	69.2	4	10.3	
**Bergmans CC**	2001	175	B, Pl, Sr	11	78	24	30.8	6	7.7	
**Blair**	1991	176	Sr	4	130	37	28.5	7	5.4	
**Bonten CC**	1994	177	B, Sr		21	0.5	0.0	0.5	0.0	
**Camus**	2005	178, 200	B, Pl, Sr		126	30	23.8	8	6.3	2
**Ferrer**	1994	179	B, Pl, Sr	12.6	41	10	24.4	2	4.9	2
**Gastinne**	1992	180	Pl, Sr		225	34	15.1	10	4.4	2
**Georges**	1994	181	B, Pl, Tr, Sr		33	15	45.5	1	3.0	
**Hammond**	1994	182	Pl, Tr	5.9	33	1	3.0	3	9.1	
**Jacobs**	1992	183	Sr		43	4	9.3	0.5	0.0	
**Karvouniaris**	2015	184	Pl	9	84	25	29.8	4	4.8	
**Korinek**	1993	185	B, Pl, Sr	15	60	25	41.7	16	26.7	
**Laggner**	1994	186	Pl, Sr	19.9	34	4	11.8	0.5	0.0	
**Langlois-Karaga**	1995	187	Pl, Tr, Sr		50	28	56.0	15	30.0	
**Palomar**	1997	188	Tr, Sr	6.4	42	21	50.0	8	19.0	
**Palomar Ctx**	1997	188	Tr, Sr		46	14	30.4	3	6.5	
**Quinio**	1995	189	Pl, Tr, Sr	9	72	37	51.4	16	22.2	
**Rocha**	1992	190	Pl, Sr	13	54	25	46.3	15	27.8	
**Rolando**	1993	191	L, Sr	7.3	31	11	35.5	1	3.2	
**Sanchez-Garcia**	1998	192	Pl, Sr	10	140	57	40.7	7	5.0	6
**Stoutenbeek '07**	2007	193	Tr, Sr	8	200	46	23.0	40	20.0	
**Ulrich**	1989	194	Sr	7.8	52	26	50.0	5	9.6	
**Unertl**	1987	195	Sr	11	20	9	45.0	5	25.0	
**Verwaest** [c]	1997	196	Sr	19	185	40	21.6	9	4.9	5
**Wiener**	1995	197	US, B, Pl, Sr	10	31	8	25.8	4	12.9	2
**Winter CC**	1992	198	B, Sr	8	92	17	18.5	1	1.1	

Table S4 (continued): *S. aureus* VAP data: studies of SDD [a]

Author	Year	Ref	Notes [b]	MVD	Patients (n)	VAP (n)	VAP %	S aureus (n)	S aureus %	MRSA (n)
**intervention groups**										
**Abele-Horn**	1997	173	Tr, Sr	13	58	13	22.4	9	15.5	
**Bergmans**	2001	175	B, Sr	10	87	9	10.3	3	3.4	
**Blair**	1991	176	Sr	5	126	11	8.7	1	0.8	
**Bonten TAP**	1994	177	B, Sr		22	0.5	0.0	0.5	0.0	
**Camus PT**	2005	178, 200	B, Sr		130	15	11.5	6	4.6	5
**Camus PT&MCh**	2005	178, 200	B, Sr		129	10	7.8	1	0.8	1
**Ferrer**	1994	179	B, Sr	13.5	39	7	17.9	3	7.7	3
**Gastinne**	1992	180	Sr		220	26	11.8	15	6.8	6
**Georges**	1994	181	B, Tr, Sr		31	3	9.7	0.5	0.0	
**Hammond**	1994	182	Tr	6.5	39	6	15.4	4	10.3	
**Jacobs**	1992	183	Sr		36	0.5	0.0	0.5	0.0	
**Karvouniaris**	2015	184		13.5	84	14	16.7	5	6.0	
**Koeman-Col**	2006	164	Sr	8.5	128	16	13	5	3.8	
**Korinek**	1993	185	B, Tr, Sr	14	63	15	23.8	9	14.3	
**Laggner**	1994	186	Sr	15.8	33	1	3.0	0.5	0.0	
**Langlois-Karaga**	1995	187	Tr, Sr		47	14	29.8	5	10.6	
**Palomar_1**	1997	188	Tr, Sr	10.8	41	7	17.1	5	12.2	
**Quinio**	1995	189	Tr, Sr	9.1	76	19	25.0	9	11.8	
**Rocha**	1992	190	Tr, Sr	13	47	7	14.9	5	10.6	
**Rolando**	1993	191	L, Sr	9	28	8	28.6	2	7.1	
**Sanchez-Garcia**	1998	192	Sr	9	131	38	29.0	5	3.8	5
**Stoutenbeek '07**	2007	193	Tr, Sr	9	201	19	9.5	18	9.0	
**Ulrich**	1989	194	Sr	10.7	48	7	14.6	2	4.2	
**Verwaest OA [c]**	1997	196	Sr	22	193	22	11.4	6	3.1	4
**Verwaest PTA [c]**	1997	196	Sr	22	200	31	15.5	9	4.5	5
**Wiener**	1995	197	US, B, Sr	10	30	8	26.7	1	3.3	0
**Winter**	1992	198	B, Sr	6	91	3	3.3	0.5	0.0	

Footnotes

a. Treatment abbreviations

- Topical antibiotic components include; topical polymyxin; topical tobramycin; topical amphotericin; topical gentamicin; topical ciprofloxacin, topical vancomycin; topical nystatin; topical neomycin; topical nalidixic acid; topical norfloxacin; topical oflaxcin; nebulized polymyxin; nebulized saline.
- Parenteral antibiotic components include; Amoycillin-clavulinate; systemic ampicillin; systemic Cefuroxime; systemic cefotaxime; systemic ciprofloxacin; systemic vancomycin; systemic ciprofloxacin; systemic trimethoprim; systemic oflaxcin; systemic ceftazidime.

b. Notes

Pl – topical placebo used to achieve observe binding

Tr – Data originating from a study for which the majority of ICU admission were for trauma

L - Data originating from a study for which all patients had severe liver disease or transplantation.

US - Data originating from a study based in the United States of America or Canada

B – VAP diagnosis based on bronchoscopic based methods for sampling.Sr - These studies were derived from the following source systematic reviews

Sr - These studies were cited in the following source systematic reviews.

- Liberati A, D'Amico R, Pifferi S, Torri V, Brazzi L, Parmelli E: Antibiotic prophylaxis to reduce respiratory tract infections and mortality in adults receiving intensive care. *Cochrane Database Syst Rev* 2009, 4.
- Pileggi C, Bianco A, Flotta D, Nobile CG, Pavia M. Prevention of ventilator-associated pneumonia, mortality and all intensive care unit acquired infections by topically applied antimicrobial or antiseptic agents: a meta-analysis of randomized controlled trials in intensive care units. *Crit Care* 2011; 15:R155.
- Silvestri L, van Saene HKF, Casarin A, Berlot G, Gullo A. 2008. Impact of selective decontamination of the digestive tract on carriage and infection due to Gram-positive and Gram-negative bacteria. A systematic review of randomised controlled trials. *Anaesth. Intensive Care* 36:324–338.
- Chan EY, Ruest A, Meade MO, Cook DJ. Oral decontamination for prevention of pneumonia in mechanically ventilated adults: systematic review and meta-analysis. *BMJ.* 2007; 334:889–900.

c. Note, the number of MRSA strains in the groups of the study by Verwaest [196] is calculated using the proportion of MRSA (55%) among the overall numbers of isolates from all sites of infection in Table 6 of this publication.

Table S5: *S. aureus* bacteremia data: all studies

author	Year	Ref	Notes	Patients (n)	S aureus bacteremia (n)	S aureus bacteremia %
**Observational studies**						
A'court	1993	1	Tr	150	5	3.3
Cade	1993	16		98	1	1.0
Craven-medical	1988	23	US	526	4	0.8
Craven-surgical	1988	23	US	799	10	1.3
Ertugrul	2006	26	Tr	100	9	9.0
Kollef '97_all	1997	54	US	680	6	0.9
Kunac	2014	56	Tr, US	206	6	2.9
Magnason	2008	60		280	1	0.4
Reusser	1989	83		40	1	2.5
Urli	2002	99		178	12	6.7
**Non antibiotic and anti-septic studies**						
Holzapfel_C_93	1993	121		149	6	4.0
Holzapfel_C_99	1999	122		200	2	1.0
Holzapfel_I_99	1999	122		199	2	1.0
Camus MCh	2005	178, 200		130	1	0.8
Fourrier'00	2000	161		30	1	3.3
Fourrier'05	2005	162	Pl	114	0.5	0.0
Fourrier'00	2000	161		30	0.5	0.0
Fourrier'05	2005	162	Pl	114	1	0.9

Table S5: *S. aureus* bacteremia data: all studies (continued)

author	Year	Ref	Notes	Patients (n)	S aureus bacteremia (n)	S aureus bacteremia %
**SDD studies – control groups**						
Camus	2005	178, 200		126	1	0.8
Ferrer	1994	179	Pl	41	2	4.9
Jacobs	1992	183		43	0.5	0.0
Laggner	1994	186	Pl	34	0.5	0.0
Quinio	1995	189	Tr, Pl	72	4	5.6
Rocha	1992	190	Tr, Pl	54	3	5.6
Rolando	1993	191	L	31	3	9.7
Stoutenbeek '07	2007	193	Tr	200	5	2.5
Verwaest	1997	196		185	3	1.6
**SDD studies – intervention groups**						
Camus PT	2005	178, 200		130	3	2.3
Camus MCh&PT	2005	178, 200		129	2	1.6
Ferrer	1994	179	Pl	39	0.5	0.0
Jacobs	1992	183		36	0.5	0.0
Laggner	1994	186	Pl	33	1	3.0
Quinio	1995	189	Tr, Pl	76	8	10.5
Rocha	1992	190	Tr, Pl	47	2	4.3
Rolando	1993	191	L	28	0.5	0.0
Stoutenbeek '07	2007	193	Tr	201	8	4.0
Verwaest OA	1997	196		193	7	3.6
Verwaest PTA	1997	196		200	10	5.0

References

S1. A'Court CH, Garrard CS, Crook D, Bowler I, Conlon C, Peto T, Anderson E: Microbiological lung surveillance in mechanically ventilated patients, using non-directed bronchial lavage and quantitative culture. Q J Med. 1993;86:635-48.

S2. Alvarez-Lerma F, ICU-acquired Pneumonia Study Group. Modification of empiric antibiotic treatment in patients with pneumonia acquired in the intensive care unit. *Intens Care Med.* 1996;22(5):387-94.

S3. Antonelli M, Moro ML, Capelli O, De Blasi RA, D'Errico RR, Conti G, Bufi M, Gasparetto A: Risk factors for early onset pneumonia in trauma patients. Chest. 1994;105:224-228

S4. Apostolopoulou E, Bakakos P, Katostaras T, Gregorakos L: Incidence and risk factors for ventilator-associated pneumonia in 4 multidisciplinary intensive care units in Athens, Greece. Respir Care. 2003;48: 681-688.

S5. Baker AM, Meredith JW, Haponik EF. Pneumonia in intubated trauma patients. Microbiology and outcomes. *Am J Respir Crit Care Med*. 1996;153:343-9.

S6. Bekaert M, Timsit JF, Vansteelandt S, Depuydt P, Vésin A, Garrouste-Orgeas M, Decruyenaere J, Clec'h C, Azoulay E, Benoit D. Attributable mortality of ventilator-associated pneumonia: a reappraisal using causal analysis. American journal of respiratory and critical care medicine. 2011;184(10):1133-9.

S7. Bercault N, Boulain T: Mortality rate attributable to ventilator-associated nosocomial pneumonia in an adult intensive care unit: a prospective case-control study. Crit Care Med. 2001;29:2303-2309

S8. Berrouane Y, Daudenthun I, Riegel B, Emery MN, Martin G, Krivosic R, Grandbastien B. Early onset pneumonia in neurosurgical intensive care unit patients. J Hosp Infect. 1998;40(4):275-80.

S9. Bochicchio GV, Joshi M, Bochicchio K, Tracy K, Scalea TM: A time-dependent analysis of intensive care unit pneumonia in trauma patients. J Trauma. 2004;56:296-301.

S10. Bonten MJ, Gaillard CA, van Tiel FH, Smeets HG, van der Geest S, Stobberingh EE: The stomach is not a source for colonization of the upper respiratory tract and pneumonia in ICU patients. Chest. 1994;105(3):878-84.

S11. Boots RJ, Phillips GE, George N, Faoagali JL: Surveillance culture utility and safety using low-volume blind bronchoalveolar lavage in the diagnosis of ventilator- associated pneumonia. Respirology. 2008;13:87-96

S12. Bornstain C, Azoulay E, De Lassence A, Cohen Y, Costa MA, Mourvillier B, Descorps-Declere A, Garrouste-Orgeas M, Thuong M, Schlemmer B, Timsit JF: Sedation, sucralfate, and antibiotic use are potential means for protection against early-onset ventilator-associated pneumonia. Clin Infect Dis. 2004;38(10):1401-8.

S13. Braun SR, Levin AB, Clark KL. Role of corticosteroids in the development of pneumonia in mechanically ventilated head-trauma victims. Crit Care Med 1986;14:198-201

S14. Bregeon F, Papazian L, Visconti A, Gregoire R, Thirion X, Gouin F: Relationship of microbiologic diagnostic criteria to morbidity and mortality in patients with ventilator-associated pneumonia. JAMA. 1997;277: 655-662

S15. Bronchard R, Albaladejo P, Brezac G, et al. Early onset pneumonia: risk factors and consequences in head trauma patients. *Anesthesiology* 2004;100:234-9.

S16. Cade JF, McOwat E, Siganporia R, Keighley C, Presneill J, Sinickas V: Uncertain relevance of gastric colonization in the seriously ill. Intensive Care Med. 1992;18:210-217

S17. Cavalcanti M, Ferrer M, Ferrer R, Morforte R, Garnacho A, Torres A: Risk and prognostic factors of ventilator-associated pneumonia in trauma patients. Crit Care Med. 2006;34:1067-1072

S18. Cendrero JA, Solé-Violán J, Benitez AB, Catalán JN, Fernández JA, Santana PS, de Castro FR: Role of different routes of tracheal colonization in the development of pneumonia in patients receiving mechanical ventilation. Chest. 1999;116:462-470

S19. Chaari A, El Habib M, Ghdhoun H, Algia NB, Chtara K, Hamida CB, Chelly H, Bahloul M, Bouaziz M. Does low-dose hydrocortisone therapy prevent ventilator-associated pneumonia in trauma patients?. Am J Therap. 2015;22(1):22-8.

S20. Chastre J, Trouillet JL, Vuagnat A, Joly-Guillou ML, Clavier H, Dombret MC, Gibert C: Nosocomial pneumonia in patients with acute respiratory distress syndrome. Am J Respir Crit Care Med. 1998;157:1165-1172

S21. Chevret S, Hemmer M, Carlet J: Incidence and risk factors of pneumonia acquired in intensive care units. Results from a multicenter prospective study on 996 patients. European Cooperative Group on Nosocomial Pneumonia. Intensive Care Med. 1993;19:256-264

S22. Cook A, Norwood S, Berne J: Ventilator-associated pneumonia is more common and of less consequence in trauma patients compared with other critically ill patients. J Trauma Acute Care Surg. 2010;69(5):1083-91.

S23. Craven DE, Kunches LM, Lichtenberg DA, Kollisch NR, Barry MA, Heeren TC, McCabe WR: Nosocomial infection and fatality in medical and surgical intensive care unit patients. Arch Intern Med. 1988;148:1161-1168

S24. Daschner F, Kappstein I, Schuster F, Scholz R, Bauer E, Jooβens D, Just H: Influence of disposable ('Conchapak') and reusable humidifying systems on the incidence of ventilation pneumonia. J Hosp Infect. 1988;11:161-168

S25. De Latorre FJ, Pont T, Ferrer A, Rosselló J, Palomar M, Planas M: Pattern of tracheal colonization during mechanical ventilation. Am J Respir Crit Care Med. 1995;152:1028-1033

S26. Ertugrul BM, Yildirim A, Ay P, Oncu S, Cagatay A, Cakar N, Ertekin C, Ozsut H, Eraksoy H, Calangu S. Ventilator-associated pneumonia in surgical emergency intensive care unit. Saudi Med J. 2006;27(1):52-7.

S27. Evans HL, Zonies DH, Warner KJ, Bulger EM, Sharar SR, Maier RV, Cuschieri J. Timing of intubation and ventilator-associated pneumonia following injury. Arch Surg. 2010;145(11):1041-6.

S28. Ewig S, Torres A, El-Ebiary M, Fàbregas N, Hernandez C, Gonzalez J, Nicolas JM, Soto L: Bacterial colonization patterns in mechanically ventilated patients with traumatic and medical head injury. Incidence, risk factors, and association with ventilator-associated pneumonia. Am J Respir Crit Care Med. 1999;159:188-198

S29. Fagon JY, Chastre J, Domart Y, Trouillet JL, Pierre J, Darne C, Gibert C: Nosocomial pneumonia in patients receiving continuous mechanical ventilation. Prospective analysis of 52 episodes with use of a protected specimen brush and quantitative culture techniques. *Am Rev Respir Dis* 1989;139:877-884.

S30. Gacouin A, Barbarot N, Camus C, Salomon S, Isslame S, Marque S, Lavoué S, Donnio PY, Thomas R, Le Tulzo Y. Late-onset ventilator-associated pneumonia in nontrauma intensive care unit patients. Anesth Analg. 2009;109(5):1584-90.

S31. Garrouste-Orgeas M, Chevret S, Arlet G, Marie O, Rouveau M, Popoff N, Schlemmer B: Oropharyngeal or gastric colonization and nosocomial pneumonia in adult intensive care unit patients. A prospective study based on genomic DNA analysis. Am J Respir Crit Care Med. 1997;156(5):1647-56.

S32. George DL, Falk PS, Wunderink RG, Leeper Jr KV, Meduri GU, Steere EL, Glen Mayhall C: Epidemiology of ventilator-acquired pneumonia based on protected bronchoscopic sampling. Am J Respir Crit Care Med. 1998;158:1839-1847

S33. Georges H, Leroy O, Guery B, Alfandari S, Beaucaire G: Predisposing factors for nosocomial pneumonia in patients receiving mechanical ventilation and requiring tracheotomy. Chest. 2000;118:767–774.

S34. Giard M, Lepape A, Allaouchiche B, Guerin C, Lehot JJ, Robert MO, Vanhems P: Early-and late-onset ventilator-associated pneumonia acquired in the intensive care unit: comparison of risk factors. *J Crit Care* 2008, 23:27-33.

S35. Gruson D, Hilbert G, Vargas F, Valentino R, Bebear C, Allery A, Bebear C, Gbikpi-Benissan GE, Cardinaud JP: Rotation and restricted use of antibiotics in a medical intensive care unit: impact on the incidence of ventilator-associated pneumonia caused by antibiotic-resistant gram-negative bacteria. Am J Respir Crit Care Med. 2000, 162(3):837-43.

S36. Gruson D, Hilbert G, Vargas F, Valentino R, Bui N, Pereyre S, Bebear C, Bebear CM, Gbikpi-Benissan G: Strategy of antibiotic rotation: long-term effect on incidence and susceptibilities of Gram-negative bacilli responsible for ventilator-associated pneumonia. Crit Care Med. 2003;31:1908-1914.

S37. Guérin C, Girard R, Chemorin C, De Varax R, Fournier G: Facial mask noninvasive mechanical ventilation reduces the incidence of nosocomial pneumonia. Intens care Med. 1997;23(10):1024-32.

S38. Guimaraes MM, Rocco JR: Prevalence of ventilator-associated pneumonia in a university hospital and prognosis for the patients affected. *J Bras Pneumol* 2006;32:339–346.

S39. Gursel G, Aydogdu M, Nadir Ozis T, Tasyurek S. Comparison of the value of initial and serial endotracheal aspirate surveillance cultures in predicting the causative pathogen of ventilator-associated pneumonia. Scandinavian J Infect Dis 2010;42:341-346

S40. Heyland DK, Cook DJ, Schoenfeld PS, Frietag A, Varon J, Wood G: The effect of acidified enteral feeds on gastric colonization in critically ill patients: results of a multicenter randomized trial. Canadian Critical Care Trials Group. Crit Care Med. 1999;27:2399-2406

S41. Hugonnet S, Uçkay I, Pittet D Staffing level: a determinant of late-onset ventilator-associated pneumonia. Crit Care. 2007;11(4):R80

S42. Hyllienmark P, Gardlund B, Persson JO, Ekdahl K. Nosocomial pneumonia in the ICU: a prospective cohort study. *Scand J Infect Dis.* 2007;39:676-82.

S43. Ibáñez J, Peñafiel A, Marsé P, Jordá R, Raurich JM, Mata F: Incidence of gastroesophageal reflux and aspiration in mechanically ventilated patients using small-bore nasogastric tubes. J Parenteral and Enteral Nutrition. 2000;24(2):103-6.

S44. Ibrahim EH, Ward S, Sherman G, Kollef MH: A comparative analysis of patients with early-onset vs late-onset nosocomial pneumonia in the ICU setting. Chest. 2000;117:1434-1442

S45. Jacobs S, Chang RW, Lee B, Bartlett FW: Continuous enteral feeding: a major cause of pneumonia among ventilated intensive care unit patients. *JPEN J Parenter Enteral Nutr* 1990;14:353-6.

S46. Jaillette E, Nseir S: Relationship between inhaled β2-agonists and ventilator-associated pneumonia: A cohort study. Critical Care Med. 2011;39(4):725-30.

S47. Jaimes F, De La Rosa G, Gómez E, Múnera P, Ramírez J, Castrillón S. Incidence and risk factors for ventilator-associated pneumonia in a developing country Where is the difference? Respir Med. 2007;101:762–767.

S48. Jiménez P, Torres A, Rodríguez-Roisin R, de la Bellacasa JP, Aznar R, Gatell JM, Agustí-Vidal A: Incidence and etiology of pneumonia acquired during mechanical ventilation. Crit Care Med. 1989;17:882-5.

S49. Kallel H, Chelly H, Bahloul M, Ksibi H, Dammak H, Chaari A, Hamida CB, Rekik N, Bouaziz M. The effect of ventilator-associated pneumonia on the prognosis of head trauma patients. J Trauma Acute Care Surg. 2005;59(3):705-10.

S50. Kanafani ZA, Kara L, Hayek S, et al. Ventilator-associated pneumonia at a tertiary-care center in a developing country: incidence, microbiology, and susceptibility patterns of isolated microorganisms. Infect Control Hosp Epidemiol. 2003;24:864-9.

S51. Kollef MH: Ventilator-associated pneumonia. A multivariate analysis. JAMA. 1993;270:1965-70.

S52. Kollef MH, Silver P, Murphy DM, Trovillion E: The effect of late-onset ventilator-associated pneumonia in determining patient mortality. Chest. 1995;108: 1655-62.

S53. Kollef MH, Von Harz B, Prentice D, Shapiro SD, Silver P, John RS, Trovillion E: Patient transport from intensive care increases the risk of developing ventilator-associated pneumonia. Chest. 1997;112(3):765-773.

S54. Kollef MH, Chastre J, Fagon JY, François B, Niederman MS, Rello J, Torres A, Vincent JL, Wunderink RG, Go KW, Rehm C. Global prospective epidemiologic and surveillance study of ventilator-associated pneumonia due to Pseudomonas aeruginosa. Crit care med. 2014;42(10):2178-87.

S55. Koss WG, Khalili TM, Lemus JF, Chelly MM, Margulies DR, Shabot MM: Nosocomial pneumonia is not prevented by protective contact isolation in the surgical intensive care unit. Am Surg. 2001;67:1140-4.

S56. Kunac A, Sifri ZC, Mohr AM, Horng H, Lavery RF, Livingston DH: Bacteremia and Ventilator-Associated Pneumonia: A Marker for Contemporaneous Extra-Pulmonic Infection. Surg Infect. 2014;15:77-83.

S57. Lepelletier D, Roquilly A, Mahe PJ, Loutrel O, Champin P, Corvec S, Naux E, Pinaud M, Lejus C, Asehnoune K. Retrospective analysis of the risk factors and pathogens associated with early-onset ventilator-associated pneumonia in surgical-ICU head-trauma patients. J Neurosurg Anesthesiol. 2010;22(1):32-7.

S58. Luna CM, Blanzaco D, Niederman MS, et al Resolution of ventilator-associated pneumonia: prospective evaluation of the clinical pulmonary infection score as an early clinical predictor of outcome. Crit Care Med 2003;31:676-682

S59. Luyt CE, Guérin V, Combes A, Trouillet JL, Ayed SB, Bernard M, Gibert C, Chastre J: Procalcitonin kinetics as a prognostic marker of ventilator-associated pneumonia. Am J Respir Crit Care Med. 2005;171:48-53.

S60. Magnason S, Kristinsson KG, Stefansson T, Erlendsdottir H, Jonsdottir K, Kristjansson M, Gudmundsson S: Risk factors and outcome in ICU- acquired infections. Acta Anaesthesiologica Scandinavica. 2008;52:1238-1245

S61. Magret M, Amaya-Villar R, Garnacho J, Lisboa T, Diaz E, DeWaele J, Deja M, Manno E, Rello J, EU-VAP/CAP Study Group: Ventilator-associated pneumonia in trauma patients is associated with lower mortality: results from EU-VAP study. J Trauma Acute Care Surg. 2010;69(4):849-854.

S62. Mahul P, Auboyer C, Jospe R, Ros A, Guerin C, el Khouri Z, Galliez M, Dumont A, Gaudin O: Prevention of nosocomial pneumonia in intubated patients respective role of mechanical subglottic secretions drainage and stress ulcer prophylaxis. Intensive Care Med. 1992;18:20-25

S63. Makris D, Manoulakas E, Komnos A, Papakrivou E, Tzovaras N, Hovas A, Zintzaras E, Zakynthinos E. Effect of pravastatin on the frequency of ventilator-associated pneumonia and on intensive care unit mortality: open-label, randomized study. Crit care med. 2011;39(11):2440-6.

S64. Markowicz P, Wolff M, Djedaini K, Cohen Y, Chastre J, Delclaux C: Multicenter prospective study of ventilator-associated pneumonia during acute respiratory distress syndrome. Incidence, prognosis, and risk factors. ARDS Study Group. Am J Respir Crit Care Med. 2000;161:1942-8.

S65. Memish ZA, Cunningham G, Oni GA, et al The incidence and risk factors of ventilator-associated pneumonia in a Riyadh hospital. Infect Control Hosp Epidemiol 2000;21:271-273

S66. Michel F, Franceschini B, Berger P, Arnal JM, Gainnier M, Sainty JM, Papazian L. Early antibiotic treatment for BAL-confirmed ventilator-associated pneumonia: a role for routine endotracheal aspirate culturesChest. 2005;127(2):589-97.

S67. Moine P, Timsit JF, De Lassence A, Troché G, Fosse JP, Alberti C, Cohen Y: Mortality associated with late-onset pneumonia in the intensive care unit: results of a multi-center cohort study. Intensive Care Med. 2002;28:154-163

S68. Myny D, Depuydt P, Colardyn F, Blot S: Ventilator-associated pneumonia in a tertiary care ICU analysis of risk factors for acquisition and mortality. Acta Clin Belg. 2005;60:114-121.

S69. Nguile-Makao M, Zahar JR, Français A, Tabah A, Garrouste-Orgeas M, Allaouchiche B, Goldgran-Toledano D, Azoulay E, Adrie C, Jamali S, Clec'h C. Attributable mortality of ventilator-associated pneumonia: respective impact of main characteristics at ICU admission and VAP onset using conditional logistic regression and multi-state models. Intens care med. 2010;36(5):781-9.

S70. Nielsen SL, Røder B, Magnussen P, Engquist A, Frimodt-møller N. Nosocomial pneumonia in an intensive care unit in a Danish university hospital: incidence, mortality and etiology. Scand J Infect Dis. 1992;24:65-70.

S71. Noor A, Hussain SF. Risk factors associated with development of ventilator associated pneumonia. J Coll Physicians Surg Pak. 2005;15:92–95.

S72. Nseir S, Di Pompeo C, Soubrier S, Cavestri B, Jozefowicz E, Saulnier F, Durocher A: Impact of ventilator-associated pneumonia on outcome in patients with COPD. Chest. 2005;128(3):1650-1656.

S73. Papazian L, Bregeon F, Thirion X, Gregoire R, Saux P, Denis JP, Perin G, Charrel J, Dumon JF, Affray JP, Gouin F: Effect of ventilator-associated pneumonia on mortality and morbidity. Am J Respir Crit Care Med. 1996;154:91-7.

S74. Potgieter PD, Linton DM, Oliver S, Forder AA: Nosocomial infections in a respiratory intensive care unit. Crit Care Med. 1987;15:495-498

S75. Raineri E, Crema L, Dal Zoppo S, Acquarolo A, Pan A, Carnevale G, Albertario F, Candiani A. Rotation of antimicrobial therapy in the intensive care unit: impact on incidence of ventilator-associated pneumonia caused by antibiotic-resistant Gram-negative bacteria. European journal of clinical microbiology & infectious diseases. 2010;29(8):1015-24.

S76. Ramirez P, Lopez-Ferraz C, Gordon M, Gimeno A, Villarreal E, Ruiz J, Menendez R, Torres A. From starting mechanical ventilation to ventilator-associated pneumonia, choosing the right moment to start antibiotic treatment. *Crit Care* 2016;20(1):169.

S77. Rello J, Quintana E, Ausina V, Castella J, Luquin M, Net A, Prats G: Incidence, etiology, and outcome of nosocomial pneumonia in mechanically ventilated patients. Chest. 1991;100:439-444

S78. Rello J, Ausina V, Ricart M, Puzo C, Net A, Prats G. Nosocomial pneumonia in critically ill comatose patients: need for a differential therapeutic approach. European Respiratory Journal. 1992;5(10):1249-53.

S79. Rello J, Sonora R, Jubert P, et al. Pneumonia in intubated patients: role of respiratory airway Care *Am J Respir Crit Care Med* 1996;154:111-5.

S80. Rello J, Ollendorf DA, Oster G, et al. Epidemiology and outcomes of ventilator-associated pneumonia in a large US database. Chest 2002;122:2115-2121

S81. Rello J, Lorente C, Diaz E, et al. Incidence, etiology, and outcome of nosocomial pneumonia in ICU patients requiring percutaneous tracheotomy for mechanical ventilation. *Chest*. 2003;124:2239-2243.

S82. Resende MM, Monteiro SG, Callegari B, Figueiredo PM, Monteiro CR, Monteiro-Neto V. Epidemiology and outcomes of ventilator-associated pneumonia in northern Brazil: an analytical descriptive prospective cohort study. *BMC Infect Dis*. 2013;13(1):119.

S83. Reusser P, Zimmerli W, Scheidegger D, Marbet GA, Buser M, Gyr K: Role of gastric colonization in nosocomial infections and endotoxemia: a prospective study in neurosurgical patients on mechanical ventilation. J Infect Dis. 1989;160:414-421

S84. Rezai MS, Bagheri–Nesami M, Nikkhah A, Bayg AH. Incidence, risk factors, and outcome of ventilator-associated Pneumonia in 18 hospitals of Iran. Running title: ventilator-associated pneumonia in Iran. *Int J Adv Biotech Res.* 2016;7(3):936-46.

S85. Rincón-Ferrari MD, Flores-Cordero JM, Leal-Noval SR, Murillo-Cabezas F, Cayuelas A, Muñoz-Sánchez MA, Sánchez-Olmedo JI: Impact of ventilator-associated pneumonia in patients with severe head injury. J Trauma Acute Care Surg. 2004;57(6):1234-40.

S86. Rodrigues PM, Neto C, Santos LR, Knibel MF. Ventilator-associated pneumonia: epidemiology and impact on the clinical evolution of ICU patients. *J Brasileiro de Pneumologia.* 2009;35(11):1084-91.

S87. Rodriguez JL, Gibbons KJ, Bitzer LG, Dechert RE, Steinberg SM, Flint LM: Pneumonia: incidence, risk factors, and outcome in injured patients. J Trauma. 1991;31: 907-12.

S88. Ruiz-Santana S, Garcia Jimenez A, Esteban A, et al. ICU pneumonias: a multi-institutional study. *Crit Care Med*. 1987;15:930-932.

S89. Salahuddin N, Zafar A, Sukhyani L, et al. Reducing ventilator-associated pneumonia rates through a staff education programme. *J Hosp Infect* 2004; 57: 223-7.

S90. Salata RA, Lederman MM, Shlaes DM, Jacobs MR, Eckstein E, Tweardy D, Toossi Z, Chmielewski R, Marino J, King CH: Diagnosis of nosocomial pneumonia in intubated, intensive care unit patients. Am Rev Respir Dis. 1987;135:426-432

S91. Shahin J, Bielinski M, Guichon C, Flemming C, Kristof AS Suspected ventilator-associated respiratory infection in severely ill patients: a prospective observational study. Crit Care 2013;17(5): R251

S92. Sofianou DC, Constandinidis TC, Yannacou M, Anastasiou H, Sofianos E: Analysis of risk factors for ventilator-associated pneumonia in a multidisciplinary intensive care unit. *Eur J Clin Microbiol Infect Dis* 2000, 19:460-463.

S93. Stéphan F, Mabrouk N, Decailliot F, Delclaux C, Legrand P: Ventilator-associated pneumonia leading to acute lung injury after trauma: importance of *Haemophilus influenzae*. Anesthesiology. 2006;104: 235-41.

S94. Tan X, Zhu S, Yan D, Chen W, Chen R, Zou J, Yan J, Zhang X, Farmakiotis D, Mylonakis E. Candida spp. airway colonization: A potential risk factor for Acinetobacter baumannii ventilator-associated pneumonia. *Med Mycol*. 2016:myw009.

S95. Tejada Artigas AT, Dronda SB, Vallés EC, Marco JM, Usón MC, Figueras P, Suarez FJ, Hernandez A: Risk factors for nosocomial pneumonia in critically ill trauma patients. Crit Care Med. 2001;29:304-9.

S96. Timsit JF, Chevret S, Valcke J, Misset B, Renaud B, Goldstein FW, Vaury P, Carlet J: Mortality of nosocomial pneumonia in ventilated patients: influence of diagnostic tools. Am J Respir Crit Care Med. 1996;154:116-23.

S97. Torres A, Aznar R, Gatell JM, Jiménez P, González J, Ferrer A, Celis R, Rodriguez-Roisin R: Incidence, risk, and prognosis factors of nosocomial pneumonia in mechanically ventilated patients. Am Rev Respir Dis. 1990;142:523-8.

S98. Trouillet JL, Chastre J, Vuagnat A, Joly-Guillou ML, Combaux D, Dombret MC, Gibert C: Ventilator-associated pneumonia caused by potentially drug-resistant bacteria. Am J Respir Crit Care Med. 1998;157(2):531-9.

S99. Urli T, Perone G, Acquarolo A, Zappa S, Antonini B, Ciani A: Surveillance of infections acquired in intensive care: usefulness in clinical practice. *J Hosp Infect* 2002, 52:130-5.

S100. Valles J, Pobo A, Garcia-Esquirol O, Mariscal D, Real J, Fernández R. Excess ICU mortality attributable to ventilator-associated pneumonia: the role of early vs late onset. *Intensive care medicine*, 2007;33(8):1363-1368.

S101. Vanhems P, Bénet T, Voirin N, Januel JM, Lepape A, Allaouchiche B, Argaud L, Chassard D, Guérin C. Early-onset ventilator-associated pneumonia incidence in intensive care units: a surveillance-based study. BMC Infect Dis. 2011;11(1):236.

S102. Verhamme KM, De Coster W, De Roo L, De Beenhouwer H, Nollet G, Verbeke J, Demeyer I, Jordens P: Pathogens in early-onset and late-onset intensive care unit–acquired pneumonia. Infection Control Hospital Epidemiol. 2007;28(4):389-397.

S103. Violan JS, Sanchez-Ramirez C, Mujica AP, Cendrero JC, Fernandez JA, de Castro FR: Impact of nosocomial pneumonia on the outcome of mechanically-ventilated patients. Crit Care (Lond). 1998;2:19-23.

S104. Woske HJ, Röding T, Schulz I, Lode H: Ventilator-associated pneumonia in a surgical intensive care unit Epidemiology, etiology and comparison of three bronchoscopic methods for microbiological specimen sampling. Crit Care. 2001;5:167–173.

S105. Xie DS, Xiong W, Lai RP, Liu L, Gan XM, Wang XH, Wang M, Lou YX, Fu XY, Wang HF, Xiang H. Ventilator-associated pneumonia in intensive care units in Hubei Province, China: a multicentre prospective cohort survey. *J Hosp Infect.* 2011;78(4):284-8.

S106. Zahar JR, Nguile-Makao M, Français A, Schwebel C, Garrouste-Orgeas M, Goldgran-Toledano D, Azoulay E, Thuong M, Jamali S, Cohen Y, De Lassence A. Predicting the risk of documented ventilator-associated pneumonia for benchmarking: construction and validation of a score. Crit care med. 2009;37(9):2545-51.

S107. Acosta-Escribano J, Fernández-Vivas M, Carmona TG, Caturla-Such J, Garcia-Martinez M, Menendez-Mainer A, Sanchez-Payá J (2010) Gastric versus transpyloric feeding in severe traumatic brain injury: a prospective, randomized trial. Intensive Care Med 36:1532-1539

S108. Bonten MJ, Gaillard CA, Van der Geest S, Van Tiel FH, Beysens AJ, Smeets HG, Stobberingh EE: The role of intragastric acidity and stress ulcer prophylaxis on colonization and infection in mechanically ventilated ICU patients. A stratified, randomized, double-blind study of sucralfate versus antacids. Am J Respir Crit Care Med. 1995;152:1825-1834.

S109. Boots RJ, Phillips GE, George N, Faoagali JL. Surveillance culture utility and safety using low-volume blind bronchoalveolar lavage in the diagnosis of ventilator- associated pneumonia. Respirology. 2008;13:87-96.

S110. Combes P, Fauvage B, Oleyer C. Nosocomial pneumonia in mechanically ventilated patients, a prospective randomised evaluation of the Stericath closed suctioning system. *Intensive Care Med* 2000;26:878-82.

S111. Cook D, Guyatt G, Marshall J, et al A comparison of sucralfate and ranitidine for the prevention of upper gastrointestinal bleeding in patients requiring mechanical ventilation. Canadian Critical Care Trials Group. N Engl J Med 1998;338:791-797

S112. Daumal F, Colpart E, Manoury B, Mariani M, Daumal M. Changing heat and moisture exchangers every 48 hours does not increase the incidence of nosocomial pneumonia. Infection Control & Hospital Epidemiology. 1999;20(5):347-9.

S113. Djedaini K, Billiard M, Mier L, Le Bourdelles G, Brun P, Markowicz P, Estagnasie P, Coste F, Boussougant Y, Dreyfuss D: Changing heat and moisture exchangers every 48 hours rather than 24 hours does not affect their efficacy and the incidence of nosocomial pneumonia. Am J Respir Crit Care Med. 1995;152(5):1562-9.

S114. Drakulovic MB, Torres A, Bauer TT, Nicolas JM, Nogué S, Ferrer M: Supine body position as a risk factor for nosocomial pneumonia in mechanically ventilated patients: a randomised trial. Lancet. 1999;354(9193):1851-1858

S115. Dreyfuss D, Djedaini K, Weber P, Brun P, Lanore JJ, Rahmani J, Coste F: Prospective study of nosocomial pneumonia and of patient and circuit colonization during mechanical ventilation with circuit changes every 48 hours versus no change. Am Rev Respir Dis. 1991;143(4 Pt 1), 738-743.

S116. Dreyfuss D, Djedaïni K, Gros I, Mier L, Le Bourdellés G, Cohen Y, Estagnasié P, Coste F, Boussougant Y: Mechanical ventilation with heated humidifiers or heat and moisture exchangers: effects on patient colonization and incidence of nosocomial pneumonia. Am J Respir Crit Care Med. 1995;151:986-92.

S117. Driks MR, Craven DE, Celli BR, et al (1987) Nosocomial pneumonia in intubated patients given sucralfate as compared with antacids or histamine type 2 blockers. The role of gastric colonization. N Engl J Med 317:1376-1382

S118. Fabian TC, Boucher BA, Croce MA, Kuhl DA, Janning SW, Coffey BC, Kudsk KA: Pneumonia and stress ulceration in severely injured patients: a prospective evaluation of the effects of stress ulcer prophylaxis. Arch Surg. 1993;128(2):185-92.

S119. Forestier C, Guelon D, Cluytens V, Guillart T, Sirot J, De champs C: Oral probiotic and prevention of *Pseudomonas aeruginosa* infections: a randomized, double-blind, placebocontrolled pilot study in intensive care unit patients. *Crit Care* 2008;12:R69.

S120. Heyland DK, Cook DJ, Schoenfeld PS, Frietag A, Varon J, Wood G The effect of acidified enteral feeds on gastric colonization in critically ill patients: results of a multicenter randomized trial. Canadian Critical Care Trials Group. Crit Care Med 1999;27:2399-2406

S121. Holzapfel L, Chevret S, Madinier G, Ohen F, Demingeon G, Coupry A, Chaudet M: Influence of long-term oro- or nasotracheal intubation on nosocomial maxillary sinusitis and pneumonia: results of a prospective, randomized, clinical trial. Crit Care Med. 1993;21:1132-1138

S122. Holzapfel L, Chastang C, Demingeon G, Bohe J, Piralla B, Coupry A: A randomized study assessing the systematic search for maxillary sinusitis in nasotracheally mechanically ventilated patients. Influence of nosocomial maxillary sinusitis on the occurrence of ventilator-associated pneumonia. Am J Respir Crit Care Med. 1999;159:695-701

S123. Kappstein I, Schulgen G, Friedrich T, Hellinger P, Benzing A, Geiger K, Daschner FD. Incidence of pneumonia in mechanically ventilated patients treated with sucralfate or cimetidine as prophylaxis for stress bleeding: bacterial colonization of the stomach. The American journal of medicine. 1991;91(2):S125-31.

S124. Kirschenbaum L, Azzi E, Sfeir T, et al. Effect of continuous lateral rotational therapy on the prevalence of ventilator-associated pneumonia in patients requiring long-term ventilatory care *Crit Care Med* 2002;30:1983-6.

S125. Kirton OC, DeHaven B, Morgan J, et al. A prospective, randomized comparison of an in-line heat moisture exchange filter and heated wire humidifiers: rates of ventilator-associated early-onset (community-acquired) or late-onset (hospital-acquired) pneumonia and incidence of endotracheal tube occlusion. *Chest* 1997;112:1055-9.

S126. Knight DJ, Gardiner D, Banks A, Snape SE, Weston VC, Bengmark S, Girling KJ: Effect of synbiotic therapy on the incidence of ventilator associated pneumonia in critically ill patients: a randomised, double-blind, placebo-controlled trial. Intensive Care Med. 2009;35:854-861.

S127. Kollef MH, Shapiro SD, Fraser VJ, Silver P, Murphy DM, Trovillion E, Hearns ML, Richards RD, Cracchilo L, Hossin L: Mechanical ventilation with or without 7-day circuit changes. A randomized controlled trial. Ann Intern Med.1995;123:168–174

S128. Kollef MH, Prentice D, Shapiro SD, Fraser VJ, Silver P, Trovillion E, Weilitz P, Von Harz BE, St. John RO. Mechanical ventilation with or without daily changes of in-line suction catheters. American journal of respiratory and critical care medicine. 1997 Aug 1;156(2):466-72.

S129. Kortbeek JB, Haigh PI, Doig C. Duodenal versus gastric feeding in ventilated blunt trauma patients: a randomized controlled trial. *J Trauma* 1999;46:992-6.

S130. Kostadima E, Kaditis AG, Alexopoulos EI, Zakynthinos E, Sfyras D. Early gastrostomy reduces the rate of ventilator-associated pneumonia in stroke or head injury patients. Eur Respir J. 2005;26(1):106-11.

S131. Lacherade JC, Auburtin M, Cerf C, Van de Louw A, Soufir L, Rebufat Y, Rezaiguia S, Ricard JD, Lellouche F, Brun-Buisson C, Brochard L: Impact of humidification systems on ventilator-associated pneumonia: a randomized multicenter trial. Am J Respir Crit Care Med. 2005;172:1276-1282

S132. Lacherade JC, De Jonghe B, Guezennec P, Debbat K, Hayon J, Monsel A, Bastuji-Garin S: Intermittent subglottic secretion drainage and ventilator-associated pneumonia A multicenter trial. Am J Respir Crit Care Med. 2010;182:910-917.

S133. Launey Y, Nesseler N, Le Cousin A, Feuillet F, Garlantezec R, Mallédant Y, Seguin P: Effect of a fever control protocol-based strategy on ventilator-associated pneumonia in severely brain-injured patients. Crit Care. 2014;18(6):1.

S134. Lorente L, Lecuona M, Málaga J, Revert C, Mora ML, Sierra A: Bacterial filters in respiratory circuits: an unnecessary cost? Crit Care Med 2003;31:2126-2130

S135. Lorente L, Lecuona M, Galván R, Ramos MJ, Mora ML, Sierra A: Periodically changing ventilator circuits is not necessary to prevent ventilator-associated pneumonia when a heat and moisture exchanger is used. Infect Control Hosp Epidemiol. 2004;25:1077-1082

S136. Lorente L, Lecuona M, Martín MM, García C, Mora ML, Sierra A: Ventilator-associated pneumonia using a closed versus an open tracheal suction system. Crit Care Med. 2005;33:115-119

S137. Lorente L, Lecuona M, Jiménez A, Mora ML, Sierra A: Tracheal suction by closed system without daily change versus open system. Intensive Care Med. 2006;32:538-44.

S138. Lorente L, Lecuona M, Jimenez A, Mora ML, Sierra A: Ventilator-associated pneumonia using a heated humidifier or a heat and moisture exchanger: a randomized controlled trial [ISRCTN88724583]. Crit Care 2006;10:R116

S139. Lorente L, Lecuona M, Jimenez A, Mora ML, Sierra: Influence of an endotracheal tube with polyurethane cuff and subglottic secretion drainage on pneumonia. Am J Respir Crit Care Med. 2007;176:1079-1083

S140. Lorente L, Lecuona M, Jiménez A, Lorenzo L, Roca I, Cabrera J, Llanos C, Mora ML: Continuous endotracheal tube cuff pressure control system protects against ventilator-associated pneumonia. Crit Care. 2014;18(2):1.

S141. Manzano F, Fernandez-Mondejar E, Colmenero M, Poyatos ME, Rivera R, Machado J, Catalan I, Artigas A: Positive-end expiratory pressure reduces incidence of ventilator-associated pneumonia in nonhypoxemic patients. Crit Care Med: 2008;36(8):2225-31.

S142. Martin C, Perrin G, Gevaudan MJ, Saux P, Gouin F. Heat and moisture exchangers and vaporizing humidifiers in the intensive care unit. Chest. 1990;97(1):144-9.

S143. Morrow LE, Kollef MH, Casale TB: Probiotic prophylaxis of ventilator-associated pneumonia: a blinded, randomized, controlled trial. Am J Respir Crit Care Med. 2010;182:1058-1064

S144. Nseir S, Zerimech F, Fournier C, Lubret R, Ramon P, Durocher A, Balduyck M: Continuous control of tracheal cuff pressure and microaspiration of gastric contents in critically ill patients. Am J Respir Crit Care Med. 2011;184(9):1041-7.

S145. Pickworth KK, Falcone RE, Hoogeboom JE, et al Occurrence of nosocomial pneumonia in mechanically ventilated trauma patients: a comparison of sucralfate and ranitidine. Crit Care Med 1993;21:1856-1862

S146. Pneumatikos I, Konstantonis D, Tsagaris I, Theodorou V, Vretzakis G, Danielides V, Bouros D: Prevention of nosocomial maxillary sinusitis in the ICU: the effects of topically applied alpha-adrenergic agonists and corticosteroids. Intensive Care Med. 2006;32:532-537

S147. Prod'hom G, Leuenberger P, Koerfer J, Blum A, Chiolero R, Schaller MD, Perret C, Spinnler O, Blondel J, Siegrist H, Saghafi L: Nosocomial pneumonia in mechanically ventilated patients receiving antacid, ranitidine, or sucralfate as prophylaxis for stress ulcer. A randomized controlled trial. Ann Intern Med. 1994;120:653-62.

S148. Reignier J, Mercier E, Le Gouge A, Boulain T, Desachy A, Bellec F, Lascarrou JB: Effect of Not Monitoring Residual Gastric Volume on Risk of Ventilator-Associated Pneumonia in Adults Receiving Mechanical Ventilation and Early Enteral Feeding. A Randomized Controlled Trial. *JAMA* 2013, 309;249-256.

S149. Rumbak MJ, Truncale T, Newton MN, Adams B, Hazard P. A Prospective, Randomized Study Comparing Early Versus Delayed Percutaneous Tracheostomy In Critically Ill Medical Patients Requiring Prolonged Mechanical Ventilation. Chest. 2000;118(4):97S-8S.

S150. Ryan P, Dawson J, Teres D, Celoria G, Navab F: Nosocomial pneumonia during stress ulcer prophylaxis with cimetidine and sucralfate. Arch Surg. 1993;128(12):1353-7.

S151. Smulders K, van der Hoeven H, Weers-Pothoff I, Vandenbroucke-Grauls C A randomized clinical trial of intermittent subglottic secretion drainage in patients receiving mechanical ventilation. Chest 2002;121:858-862

S152. Staudinger T, Bojic A, Holzinger U, Meyer B, Rohwer M, Mallner F, Locker GJ Continuous lateral rotation therapy to prevent ventilator-associated pneumonia Crit Care Med 2010;38(2):486-490

S153. Thomachot L, Viviand X, Arnaud S, Boisson C, Martin CD: Comparing two heat and moisture exchangers, one hydrophobic and one hygroscopic, on humidifying efficacy and the rate of nosocomial pneumonia. Chest. 1998;114:1383-1389

S154. Thomachot L, Leone M, Razzouk K, Antonini F, Vialet R, Martin C: Do the components of heat and moisture exchanger filters affect humidifying efficacy and the incidence of nosocomial pneumonia? Crit Care Med. 1999;27:923–928

S155. Thomachot L, Leone M, Razzouk K, Antonini F, Vialet R, Martin C: Randomized Clinical Trial of Extended Use of a Hydrophobic Condenser Humidifier: 1 vs 7 Days. Crit Care Med. 2002;30:232-7

S156. Valencia M, Ferrer M, Farre R, Navajas D, Badia JR, Nicolas JM, Torres A: Automatic control of tracheal tube cuff pressure in ventilated patients in semirecumbent position: a randomized trial. Crit Care Med. 2007;35: 1543-9.

S157. Zeng J, Wang CT, Zhang FS, Qi F, Wang SF, Ma S, Wu TJ, Tian H, Tian ZT, Zhang SL, Qu Y. Effect of probiotics on the incidence of ventilator-associated pneumonia in critically ill patients: a randomized controlled multicenter trial. Intens care med. 2016;42(6):1018-28.

S158. Kantorova I, Svoboda P, Scheer P, Doubek J, Rehorkova D, Bosakova H, Ochmann J. Stress ulcer prophylaxis in critically ill patients: a randomized controlled trial. Hepato-gastroenterology. 2004;51(57):757-61.

S159. Ćabov T, Macan D, Husedžinović I, Škrlin-Šubić J, Bošnjak D, Šestan-Crnek S, Perić B, Kovač Z, Golubović V. The impact of oral health and 0.2% chlorhexidine oral gel on the prevalence of nosocomial infections in surgical intensive-care patients: a randomized placebo-controlled study. Wiener klinische Wochenschrift. 2010;122(13):397-404.

S160. Caruso P, Denari S, Ruiz SA, Demarzo SE, Deheinzelin D Saline instillation before tracheal suctioning decreases the incidence of ventilator-associated pneumonia. Crit Care Med 2009;37:32-38

S161. Fourrier FE, Cau-Pottier H, Boutigny M, Roussel-Delvallez M, Jourdain, Chopin C: Effects of dental plaque antiseptic decontamination on bacterial colonization and nosocomial infections in critically ill patients. Intensive Care Med. 2000;26:1239-1247

S162. Fourrier F, Dubois D, Pronnier P, Herbecq P, Leroy O, Desmettre T, Roussel-Delvallez M: Effect of gingival and dental plaque antiseptic decontamination on nosocomial infections acquired in the intensive care unit a double-blind placebo-controlled multicenter study. Crit Care Med. 2005;33:1728-1735

S163. Genuit T, Bochicchio G, Napolitano LM, McCarter RJ, Roghman MC. Prophylactic chlorhexidine oral rinse decreases ventilator-associated pneumonia in surgical ICU patients. Surg Infect (Larchmt). 2001;2:5-18.

S164. Koeman M, van der Ven AJ, Hak E, et al. Oral decontamination with chlorhexidine reduces the incidence of ventilator-associated pneumonia. Am J Respir Crit Care Med 2006;173:1348-1355

S165. Kollef MH, Afessa B, Anzueto A, Veremakis C, Kerr KM, Margolis BD, Schinner R: Silver-coated endotracheal tubes and incidence of ventilator-associated pneumonia: the NASCENT randomized trial. JAMA. 2008;300(7):805-813

S166. Lorente L, Lecuona M, Jiménez A, Palmero S, Pastor E, Lafuente N, Ramos MJ, Mora ML, Sierra A: Ventilator-associated pneumonia with or without toothbrushing a randomized controlled trial. Eur J Clin Microbiol Infect Dis. 2012;31:1-9

S167. Mori H, Hirasawa H, Oda S, Shiga H, Matsuda K, Nakamura M Oral care reduces incidence of ventilator-associated pneumonia in ICU populations. Intensive Care Med 2006;32(2):230-236

S168. Panchabhai TS, Dangayach NS, Krishnan A, Kothari VM, Karnad DR. Oropharyngeal cleansing with 0.2% chlorhexidine for prevention of nosocomial pneumonia in critically ill patients: an open-label randomized trial with 0.01% potassium permanganate as control. Chest. 2009;135(5):1150-6.

S169. Seguin P, Tanguy M , Laviolle B, Tirel O, Malledant Y: Effect of oropharyngeal decontamination by povidone-iodine on ventilator-associated pneumonia in patients with head trauma. *Crit Care Med* 2006, 34:1514-1519.

S170. Seguin P, Laviolle B, Dahyot-Fizelier C, Dumont R, Veber B, Gergaud S, Asehnoune K, Mimoz O, Donnio PY, Bellissant E, Malledant Y. Effect of oropharyngeal povidone-iodine preventive oral care on ventilator-associated pneumonia in severely brain-injured or cerebral hemorrhage patients: a multicenter, randomized controlled trial. Crit care med. 2014;42(1):1-8.

S171. Tantipong H, Morkchareonpong C, Jaiyindee S, Thamlikitkul V. Randomized controlled trial and meta-analysis of oral decontamination with 2% chlorhexidine solution for the prevention of ventilator-associated pneumonia. Infect Control Hosp Epidemiol 2008;29:131-136.

S172. Pobo A, Lisboa T, Rodriguez A, Sole R, Magret M, Trefler S, Gómez F, Rello J. A randomized trial of dental brushing for preventing ventilator-associated pneumonia. Chest 2009;136:433-439.

S173. Abele-Horn M, Dauber A, Bauernfeind A, Russwurm W, Seyfarth-Metzger I, Gleich P, Ruckdeschel G: Decrease in nosocomial pneumonia in ventilated patients by selective oropharyngeal decontamination (SOD). Intensive Care Med. 1997;23:187-95.

S174. Aerdts SJ, van Dalen R, Clasener HA, Festen J, van Lier HJ, Vollaard EJ: Antibiotic prophylaxis of respiratory tract infection in mechanically ventilated patients. A prospective, blinded, randomized trial of the effect of a novel regimen. Chest. 1991;100:783-791

S175. Bergmans DC, Bonten MJ, Gaillard CA, et al Prevention of ventilator-associated pneumonia by oral decontamination: a prospective, randomized, double-blind, placebo-controlled study. Am J Respir Crit Care Med 2001;164:382-388

S176. Blair P, Rowlands BJ, Lowry K, Webb H, Armstrong P, Smilie J Selective decontamination of the digestive tract: a stratified, randomized, prospective study in a mixed intensive care unit. Surgery 1991;110:303-309

S177. Bonten MJ, Gaillard CA, Johanson Jr WG, Van Tiel FH, Smeets HG, Van Der Geest S, Stobberingh EE. Colonization in patients receiving and not receiving topical antimicrobial prophylaxis. *Am J Respir Crit Care Med* 1994;150(5):1332-1340.

S178. Camus C, Salomon S, Bouchigny C, Gacouin A, Lavoué S, Donnio PY, Bellissant E (2014) Short-Term Decline in All-Cause Acquired Infections With the Routine Use of a Decontamination Regimen Combining Topical Polymyxin, Tobramycin, and Amphotericin B With Mupirocin and Chlorhexidine in the ICU: A Single-Center Experience. Crit Care Med 42:1121-1130

S179. Ferrer M, Torres A, Gonzalez J, Puig de la Bellacasa J, el-Ebiary M, Roca M, Gatell JM, Rodriguez-Roisin R: Utility of selective digestive decontamination in mechanically ventilated patients. Ann Intern Med. 1994;120:389-395

S180. Godard J, Guillaume C, Reverdy ME, Bachmann P, Bui-Xuan B, Nageotte A, Motin J: Intestinal decontamination in a polyvalent ICU. A double-blind study. *Intensive Care Med* 1990, 16:307-311.

S181. Georges B, Mazerolles M, Decun J-F, et al. Décontamination digestive sélective résultats d'une étude chez le polytraumatisé. Réanimation Soins Intensifs Médecin d'Urgence 1994; 3: 621-7.

S182. Hammond JM, Potgieter PD, Saunders LG. Selective decontamination of the digestive tract in multiple trauma patients-Is there a role? Results of a prospective, double-blind, randomized trial. Crit Care Med. 1994;22(1):33-9.

S183. Jacobs S, Foweraker JE, Roberts SE: Effectiveness of selective decontamination of the digestive tract (SDD) in an ICU with a policy encouraging a low gastric pH. Clin Intensive Med. 1992;3:52-58

S184. Karvouniaris M, Makris D, Zygoulis P, Triantaris A, Xitsas S, Mantzarlis K, Petinaki E, Zakynthinos E. Nebulised colistin for ventilator-associated pneumonia prevention. Eur Resp J. 2015:46:1544-1547.

S185. Korinek AM, Laisne MJ, Nicolas MH, Raskine L, Deroin V, Sanson-lepors MJ: Selective decontamination of the digestive tract in neurosurgical intensive care unit patients: a double-blind, randomized, placebo-controlled study. Crit Care Med. 1993;21:1466-73.

S186. Laggner AN, Tryba M, Georgopoulos A, Lenz K, Grimm G, Graninger W, Schneeweiss B, Druml W (1994) Oropharyngeal decontamination with gentamicin for long-term ventilated patients on stress ulcer prophylaxis with sucralfate? Wien Klin Wochenschr 106:15-19

S187. Langlois-Karaga A, Bues-Charbit M, Davignon A, Albanese J, Durbec O, Martin C, Morati N, Balansard G. Selective digestive decontamination in multiple trauma patients: cost and efficacy. Pharmacy World and Science. 1995 Jan 1;17(1):12-6.

S188. Palomar M, Alvarez-Lerma F, Jorda R, Bermejo B, Catalan Study Group of Nosocomial Pneumonia Prevention: Prevention of nosocomial infection in mechanically ventilated patients: selective digestive decontamination versus sucralfate. Clin Intens Care. 1997;8:228-235

S189. Quinio B, Albanese J, Bues-Charbit M, Viviand X, Martin C; Selective decontamination of the digestive tract in multiple trauma patients. A prospective double-blind, randomized, placebo-controlled study. Chest 1996;109:765-772

S190. Rocha LA, Martin MJ, Pita S, Paz J, Seco C, Margusino L, Villanueva R, Duran MT: Prevention of nosocomial infection in critically ill patients by selective decontamination of the digestive tract. A randomized, double blind, placebo-controlled study. Intensive Care Med. 1992;18:398-404

S191. Rolando N, Gimson A, Wade J, Philpott- Howard J, Casewell M, Williams R: Prospective controlled trial of selective parenteral and enteral antimicrobial regimen in fulminant liver failure. Hepatol. 1993;17:196-201

S192. Sanchez-Garcia M, Cambronero JA, Lopez-Diaz J, et al. Effectiveness and cost of selective decontamination of the digestive tract in critically ill intubated patients. A randomized, double-blind, placebo-controlled multicenter trial. Am Rev Respir Dis 1998; 158:908-16.

S193. Stoutenbeek CP, van Saene HKF, Little RA, Whitehead A: The effect of selective decontamination of the digestive tract on mortality in multiple trauma patients: a multicenter randomized controlled trial. Intensive Care Med. 2007;33:261-270

S194. Unertl K, Ruckdeschel G, Selbmann HK, et al; Prevention of colonization and respiratory infections in long-term ventilated patients by local antimicrobial prophylaxis. Intensive Care Med 1987;13:106-113

S195. Ulrich C, Harinck-deWeerd JE, Bakker NC, et al. Selective decontamination of the digestive tract with norfloxacin in the prevention of ICU-acquired infections: A prospective randomized study. Intensive Care Med 1989; 15: 424-31.

S196. Verwaest C, Verhaegen J, Ferdinande P, Schetz M, Van den Berghe G, Verbist L, Lauwers P: Randomized, controlled trial of selective digestive decontamination in 600 mechanically ventilated patients in a multidisciplinary intensive care unit. Crit Care Med. 1997;25:63-71

S197. Wiener J, Itokazu G, Nathan C, Kabins SA, Weinstein RA: A randomized, double-blind, placebo-controlled trial of selective digestive decontamination in a medical-surgical intensive care unit. Clin Infect Dis. 1995;20:861-867

S198. Winter R, Humphreys H, Pick A, MacGowan AP, Willatts SM, Speller DC: A controlled trial of selective decontamination of the digestive tract in intensive care and its effect on nosocomial infection. J Antimicrob Chemother. 1992;30:73-87

S199. Verhaegen J: Randomized study of selective digestive decontamination on colonization and prevention of infection in mechanically ventilated patients in the ICU. 1992. Doctor in Medical Sciences – thesis, University Hospital, Leuven, Belgium.

S200. Camus C, Sebille V, Legras A, Garo B, Renault A, Le Corre P, Donnio PY, Gacouin A, Perrotin D, Le Tulzo Y, Bellissant E. Mupirocin/chlorexidine to prevent methicillin-resistant Staphylococcus aureus infections: post hoc analysis of a placebo-controlled, randomized trial using mupirocin/chlorhexidine and polymyxin/tobramycin for the prevention of acquired infections in intubated patients. Infection. 2014;42(3):493-502.
